# *In vivo* PET classification of tau pathologies in patients with frontotemporal dementia

**DOI:** 10.1093/braincomms/fcae075

**Published:** 2024-03-01

**Authors:** Manabu Kubota, Hironobu Endo, Keisuke Takahata, Kenji Tagai, Hisaomi Suzuki, Mitsumoto Onaya, Yasunori Sano, Yasuharu Yamamoto, Shin Kurose, Kiwamu Matsuoka, Chie Seki, Hitoshi Shinotoh, Kazunori Kawamura, Ming-Rong Zhang, Yuhei Takado, Hitoshi Shimada, Makoto Higuchi

**Affiliations:** Department of Functional Brain Imaging, Institute for Quantum Medical Science, Quantum Life and Medical Science Directorate, National Institutes for Quantum Science and Technology, Chiba 263-8555, Japan; Department of Psychiatry, Kyoto University Graduate School of Medicine, Sakyo-ku Kyoto 606-8507, Japan; Department of Functional Brain Imaging, Institute for Quantum Medical Science, Quantum Life and Medical Science Directorate, National Institutes for Quantum Science and Technology, Chiba 263-8555, Japan; Department of Functional Brain Imaging, Institute for Quantum Medical Science, Quantum Life and Medical Science Directorate, National Institutes for Quantum Science and Technology, Chiba 263-8555, Japan; Department of Neuropsychiatry, Keio University School of Medicine, Tokyo 160-8582, Japan; Department of Functional Brain Imaging, Institute for Quantum Medical Science, Quantum Life and Medical Science Directorate, National Institutes for Quantum Science and Technology, Chiba 263-8555, Japan; Department of Psychiatry, Jikei University Graduate School of Medicine, Tokyo 105-8461, Japan; Department of Functional Brain Imaging, Institute for Quantum Medical Science, Quantum Life and Medical Science Directorate, National Institutes for Quantum Science and Technology, Chiba 263-8555, Japan; Department of Psychiatry, National Hospital OrganizationShimofusa Psychiatric Center, Chiba 266-0007, Japan; Department of Psychiatry, National Hospital OrganizationShimofusa Psychiatric Center, Chiba 266-0007, Japan; Department of Functional Brain Imaging, Institute for Quantum Medical Science, Quantum Life and Medical Science Directorate, National Institutes for Quantum Science and Technology, Chiba 263-8555, Japan; Department of Neuropsychiatry, Keio University School of Medicine, Tokyo 160-8582, Japan; Department of Functional Brain Imaging, Institute for Quantum Medical Science, Quantum Life and Medical Science Directorate, National Institutes for Quantum Science and Technology, Chiba 263-8555, Japan; Department of Neuropsychiatry, Keio University School of Medicine, Tokyo 160-8582, Japan; Department of Functional Brain Imaging, Institute for Quantum Medical Science, Quantum Life and Medical Science Directorate, National Institutes for Quantum Science and Technology, Chiba 263-8555, Japan; Department of Neuropsychiatry, Keio University School of Medicine, Tokyo 160-8582, Japan; Department of Functional Brain Imaging, Institute for Quantum Medical Science, Quantum Life and Medical Science Directorate, National Institutes for Quantum Science and Technology, Chiba 263-8555, Japan; Department of Psychiatry, Nara Medical University, Nara 634-8521, Japan; Department of Functional Brain Imaging, Institute for Quantum Medical Science, Quantum Life and Medical Science Directorate, National Institutes for Quantum Science and Technology, Chiba 263-8555, Japan; Department of Functional Brain Imaging, Institute for Quantum Medical Science, Quantum Life and Medical Science Directorate, National Institutes for Quantum Science and Technology, Chiba 263-8555, Japan; Department of Advanced Nuclear Medicine Sciences, Institute for Quantum Medical Science, Quantum Life and Medical Science Directorate, National Institutes for Quantum Science and Technology, Chiba 263-8555, Japan; Department of Advanced Nuclear Medicine Sciences, Institute for Quantum Medical Science, Quantum Life and Medical Science Directorate, National Institutes for Quantum Science and Technology, Chiba 263-8555, Japan; Department of Functional Brain Imaging, Institute for Quantum Medical Science, Quantum Life and Medical Science Directorate, National Institutes for Quantum Science and Technology, Chiba 263-8555, Japan; Department of Functional Brain Imaging, Institute for Quantum Medical Science, Quantum Life and Medical Science Directorate, National Institutes for Quantum Science and Technology, Chiba 263-8555, Japan; Department of Functional Neurology and Neurosurgery, Center for Integrated Human Brain Science, Brain Research Institute, Niigata University, Niigata 951-8585, Japan; Department of Functional Brain Imaging, Institute for Quantum Medical Science, Quantum Life and Medical Science Directorate, National Institutes for Quantum Science and Technology, Chiba 263-8555, Japan

**Keywords:** frontotemporal lobar degeneration, PET, florzolotau, biomarker, tauopathy

## Abstract

Frontotemporal dementia refers to a group of neurodegenerative disorders with diverse clinical and neuropathological features. *In vivo* neuropathological assessments of frontotemporal dementia at an individual level have hitherto not been successful. In this study, we aim to classify patients with frontotemporal dementia based on topologies of tau protein aggregates captured by PET with ^18^F-florzolotau (aka ^18^F-APN-1607 and ^18^F-PM-PBB3), which allows high-contrast imaging of diverse tau fibrils in Alzheimer’s disease as well as in non–Alzheimer’s disease tauopathies. Twenty-six patients with frontotemporal dementia, 15 with behavioural variant frontotemporal dementia and 11 with other frontotemporal dementia phenotypes, and 20 age- and sex-matched healthy controls were included in this study. They underwent PET imaging of amyloid and tau depositions with ^11^C-PiB and ^18^F-florzolotau, respectively. By combining visual and quantitative analyses of PET images, the patients with behavioural variant frontotemporal dementia were classified into the following subgroups: (i) predominant tau accumulations in frontotemporal and frontolimbic cortices resembling three-repeat tauopathies (*n* = 3), (ii) predominant tau accumulations in posterior cortical and subcortical structures indicative of four-repeat tauopathies (*n* = 4); (iii) amyloid and tau accumulations consistent with Alzheimer’s disease (*n* = 4); and (iv) no overt amyloid and tau pathologies (*n* = 4). Despite these distinctions, clinical symptoms and localizations of brain atrophy did not significantly differ among the identified behavioural variant frontotemporal dementia subgroups. The patients with other frontotemporal dementia phenotypes were also classified into similar subgroups. The results suggest that PET with ^18^F-florzolotau potentially allows the classification of each individual with frontotemporal dementia on a neuropathological basis, which might not be possible by symptomatic and volumetric assessments.

## Teaser

Kubota *et al*. performed PET imaging with ^18^F-florzolotau in patients with frontotemporal dementia and categorized them into subgroups based on PET-assessed tau topologies. The proposed method may allow neuropathology-based classification of living individuals with this illness, which may not be feasible based on clinical symptoms and brain structural features.

## Graphical Abstract

**Graphical Abstract:**
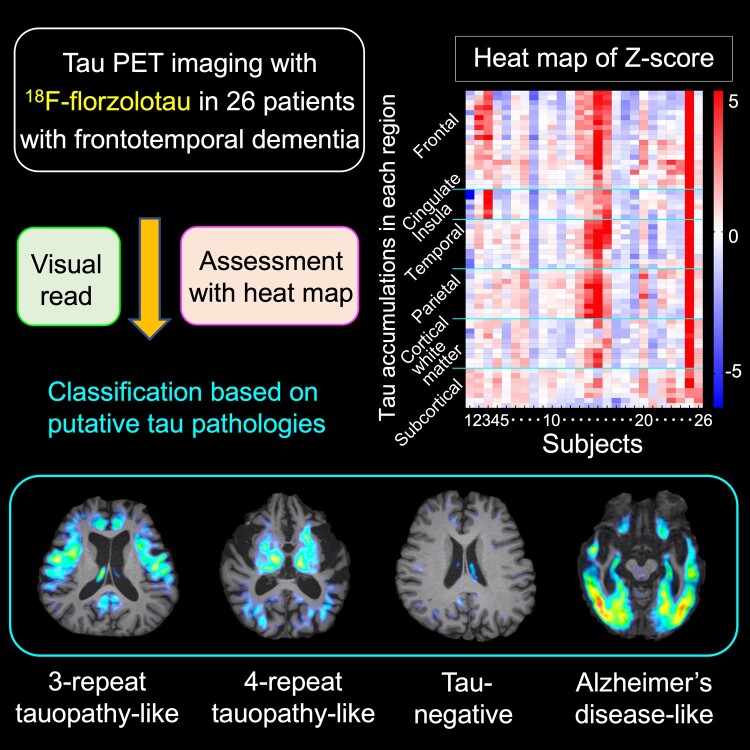

## Introduction

Frontotemporal dementia (FTD) is characterized by particular behavioural, psychiatric, speech and motor symptoms and involves progressive atrophy in the frontotemporal lobes. Clinical subtypes of FTD consist of behavioural variant FTD (bvFTD), progressive non-fluent aphasia (PNFA) and other types of primary progressive aphasia (PPA), as well as corticobasal syndrome (CBS) and progressive supranuclear palsy (PSP).^1,2^ The neuropathology of FTD is diverse and can be classified as frontotemporal lobar degenerations (FTLDs) characterized by depositions of tau proteins (FTLD-tau), transactive response DNA-binding protein 43 (TDP-43) (FTLD-TDP) and fused in sarcoma.^2^ FTLD-tau is further subcategorized into three major pathological phenotypes that include Pick’s disease, corticobasal degeneration (CBD) and PSP, along with less common forms such as argyrophilic grain dementia.^2^

Notably, there is considerable complexity in the correspondence between these clinical syndromes and molecular pathologies in patients with FTD.^2^ In fact, while bvFTD is the most common clinical subtype of FTD characterized by changes in personality and social behaviours, emotion and insight,^3^ its neuropathological backgrounds include all FTLD-tau subcategories (i.e. PSP, CBD, Pick’s disease and argyrophilic grain dementia) and FTLD-TDP,^4^ precluding the estimation of disease-associated protein subspecies based on clinical profiles.

To date, PET with specific radioligands has offered the means for visualizing tau depositions in the brains of patients with Alzheimer’s disease,^5,6^ whereas most of the PET probes, as exemplified by ^18^F-flortaucipir (^18^F-AV1451), ^18^F-MK6240 and ^18^F-THK5351, were incapable of capturing FTLD-type tau fibrils with sufficient sensitivity and specificity.^5,6^ It should be noted that tau aggregates in PSP and CBD consist of four-repeat tau isoforms (4RTs), and Pick’s disease is characterized by accumulations of three-repeat tau isoforms (3RTs), unlike Alzheimer’s disease tau pathologies that are formed by all six isoforms. The structural diversity of disease-specific tau assemblies stemming from the isoform composition and other hitherto unclarified factors gives rise to differential reactivities of the radioligands with these aggregates.

Indeed, ^18^F-flortaucipir and ^18^F-MK6240 were shown to be rather specific for Alzheimer’s disease versus FTD tau deposits and to yield mild increases of radio signals that considerably overlap with those in healthy controls in the frontotemporal subregions of amyloid-β (Aβ)-negative bvFTD, PNFA, CBS and PSP brains.^7-10^ Likewise, putative 4RT accumulations resulting from *MAPT* mutations (e.g. P301L substitution) provoked modestly or moderately enhanced retentions of these radioligands, as opposed to the profound contrast for Alzheimer’s disease–like tau aggregates involving all isoforms as a consequence of the R406W *MAPT* mutation.^11^ Although ^18^F-THK5351 yielded notable radio signals in supposedly tau-rich areas of PSP and Aβ-negative CBD brains, this compound is known to cross-react with monoamine oxidase-B, which is overexpressed in reactive astrocytes.^12^

Among these radioligands, ^11^C-PBB3 was generated to capture a wide range of tau aggregates, including those in 3RT and 4RT pathologies of non-Alzheimer’s disease type.^13^  ^11^C-PBB3 has enabled the visualization of tau depositions in patients with Alzheimer’s disease, PSP and traumatic brain injury,^14-16^ but it has only produced a relatively small dynamic range due to its high propensity for metabolic conversions. Subsequently, ^18^F-florzolotau (aka ^18^F-APN-1607 and ^18^F-PM-PBB3)^17^ was developed for high-contrast imaging of diverse tau fibrils characteristic of both Alzheimer’s disease and FTLD and has been applied to clinical PET assays by independent research groups.^17-21^ Our study of tauopathy patients with biopsy- and autopsy-confirmed tau pathologies has demonstrated that ^18^F-florzolotau is capable of detecting tau aggregates in non–Alzheimer’s disease tauopathies, such as PSP, CBD and Pick’s disease.^17^ Furthermore, ^18^F-florzolotau has clearly demonstrated characteristic topologies of tau depositions due to P301L and several other *MAPT* mutations causative of 4RT pathologies.^22^

The aim of the current study is to stratify patients with FTD based on the topology of tau accumulations assessed by PET with ^18^F-florzolotau. Four neuropathological phenotypes consisting of 3RT- and 4RT-dominant and Alzheimer’s disease–like tau pathologies and tau-negative FTD were estimated by visual read and region-wise quantification of tau PET images.

## Materials and methods

### Participants

A total of 26 patients, 15 diagnosed with bvFTD^23^ and 11 with other FTD phenotypes, were recruited from our affiliated hospitals and clinics. The latter consisted of four patients with PNFA, three with PPA,^24^ three with CBS and one with PSP with predominant frontal presentation (PSP-F).^25,26^ None of the patients were comorbid with other neuropsychiatric disorders. The sample size was determined based on previous tau PET studies of FTD and related disorders.^9,11,22,27^ The bvFTD group included one patient with autopsy-confirmed Pick’s disease (autopsy was carried out 1 year after the PET scan). Twenty age- and sex-matched healthy controls were recruited by the Institute for Quantum Medical Science, Chiba, Japan, for participation in this study. They had no history of neuropsychiatric disease, neurological injury or disease, severe medical diseases, substance abuse possibly affecting brain functions or first-degree relatives suffering from FTLD. All participants were clinically evaluated by at least one board-certified psychiatrist and one board-certified neurologist. A mini-mental state examination (MMSE) and frontal assessment battery (FAB) were conducted for all subjects. For patients with bvFTD, the severity of clinical symptoms was assessed by Neuropsychiatric Inventory (NPI) and Stereotypy Rating Inventory (SRI).^28^ Four of the patients (two with bvFTD, including the case with autopsy-confirmed Pick’s disease, one with PNFA and one with CBS) had also been included in our previous study.^17^ NPI and SRI scores for one bvFTD patient were not available. Recruitment and data collection were conducted between May 2017 and March 2020. Table 1 presents the demographic information of the participants. This study was approved by the Radiation Drug Safety Committee and the Institutional Review Board of the National Institutes for Quantum Science and Technology, Japan, and was carried out in accordance with the Code of Ethics of the World Medical Association. After a complete description of the study, written informed consent was obtained from all participants.

**Table 1:** Characteristics of subjects included in this study

	Patients (*N* = 26)			Controls (*N* = 20)			Statistics	
	*n*	Mean	SD	*n*	Mean	SD	*t*/*χ*^2^	*P*
Age (years)	26	67.6	9.4	20	65.4	6.5	*t* = 0.94	0.35
Sex (male/female)	26	9/17		20	10/10		*χ* ^2^ = 2.49	0.29
Education (years)	26	13.1	2.4	20	15.2	2.3	*t* = −2.95	0.005
Diagnosis (bvFTD/PPA/PNFA/CBS/PSP-F)	26	15/3/4/3/1						
Duration of illness (years)	26	3.8	3.5					
MMSE	26	19.3	9.2	20	28.7	1.4	*t* = −5.10	<0.001
FAB	26	8.7	4.8	20	16.6	1.2	*t* = −7.95	<0.001
NPI (bvFTD cases only)^a^	14	33.8	15.9					
SRI (bvFTD cases only)^a^	14	12.4	9.7					
Injected radioactivity for ^11^C-PiB (MBq)	26	539	43	20	518	64	*t* = 1.36	0.18
Injected radioactivity for ^18^F-florzolotau (MBq)	26	188	6	20	187	6	*t* = 0.52	0.61

^a^Data for one bvFTD patient are not available.

### MRI data acquisition and processing

All participants underwent MRI scans with a 3-T MRI scanner (MAGNETOM Verio, Siemens, Germany). Three-dimensional volumetric acquisition of a T_1_-weighted gradient echo sequence produced a gapless series of thin sagittal sections [echo time (TE)/repetition time (TR) = 1.95/2300 ms, inversion time (TI) = 900 ms, flip angle = 9°, field of view (FOV) = 250 mm, acquisition matrix = 256 × 256 and slice thickness = 1 mm].

For each T_1_-weighted image, surface-based cortical reconstruction and volumetric subcortical segmentation were performed with FreeSurfer software (version 6.0.0; http://surfer.nmr.mgh.harvard.edu/), and regions of interest (ROIs) within cerebral grey matter (GM) and white matter (WM), subcortical structures, cerebellum and brainstem were defined using FreeSurfer’s brain atlases^29-31^ for the subsequent PET analysis.

### PET data acquisition

Each participant underwent PET scans with ^11^C-PiB and ^18^F-florzolotau to quantify Aβ and tau accumulations, respectively.

Radiosynthesis of these PET ligands was carried out as described elsewhere.^32,33^ Injected radioactivities of ^11^C-PiB and ^18^F-florzolotau are shown in Table 1. For details of PET acquisition, see Supplementary Methods 1.

All PET images were corrected for scatter using a single-scatter simulation method. A head fixation device was used to minimize the subject’s head movement during the PET measurements. Motion-corrected PET images were co-registered to the corresponding individual T_1_-weighted MR images using PMOD^®^ software version 3.8 (PMOD Technologies Ltd., Zurich, Switzerland). Parametric PET images were generated by voxel-based calculation of the standardized uptake value ratio (SUVR) to the cerebellar GM (excluding the vermis) at 50–70 and 90–110 min for ^11^C-PiB and ^18^F-florzolotau, respectively.

### Data analysis

#### Evaluation of Aβ positivity

Three researchers classified all participants into Aβ-positive and Aβ-negative groups by a visual inspection of ^11^C-PiB SUVR images, based on the standard method used by the Japanese Alzheimer’s Disease Neuroimaging Initiative study.^34^ In addition, Aβ positivity was confirmed by a cortical SUVR over 1.34 on ^11^C-PiB-PET scans.^14^ We considered Aβ-positive patients to be afflicted with FTD due to Alzheimer’s disease pathologies and examined whether tau topologies in these cases followed Braak tau staging.^35^

#### Assessments of ^18^F-florzolotau-PET images

##### Visual assessments of ^18^F-florzolotau SUVR images

Three investigators (M.K., H.E. and K.T.), all experienced readers of ^18^F-florzolotau-PET data, independently assessed ^18^F-florzolotau SUVR images. Tau positivity was determined by the presence or absence of ^18^F-florzolotau accumulations in the focal frontotemporal, limbic or parietal cortices, striatum, subthalamic regions and pons, as identified in the neuropathological investigations of FTLD-tau.^2^ Each investigator performed a visual reading twice, and the second reading was used for formal visual evaluation. Patients without Aβ positivity were classified into ‘3RT-like’, ‘4RT-like’ or ‘tau-negative’. Consensus for evaluation was reached based on agreement by at least two of the three readers. Agreements among classifications by three readers and between two judgements by the same reader were examined by *κ* statistics of Fleiss and Cohen, respectively.

##### Quantitative assessments of regional ^18^F-florzolotau SUVR maps

For patients with bvFTD, we calculated *Z*-scores for ^18^F-florzolotau SUVRs in each ROI that was defined in individual MRI space using FreeSurfer’s atlases as follows:

*Z*-score = (crude individual SUVR−mean SUVR in healthy controls)/(standard deviation of SUVR in healthy controls).

Of a total of 64 regions within GM and WM of the frontal, temporal, parietal, anterior cingulate and insular cortices, striatum and midbrain, the presence of one or more regions with *Z*-score > 2 or 10 or more regions with *Z*-score > 1 was considered tau-positive, similar to previous studies.^36-38^

For Aβ-negative and tau-positive bvFTD patients, we investigated their tau topologies after controlling for brain atrophy. We applied partial volume correction (PVC) using the volume-of-interest–based Geometric Transfer Matrix method^39^ and calculated their regional *Z*-scores for each ROI using SUVRs of healthy controls corrected for partial volume effects. With reference to previous literature on the distribution patterns of 3RT and 4RT pathologies,^2,40,41^ we conceived the topology of PET tracer retentions as ‘3RT-like’ and ‘4RT-like’ according to the following criteria: of 22 regions consisting of inferior and middle pre-frontal (i.e. medial and lateral orbitofrontal, pars orbitalis, pars triangularis, pars opercularis and rostral and caudal middle frontal) GM, inferior, middle and superior temporal GM and caudate, the presence of 10 or more regions with *Z*-score > 1 was considered ‘3RT-like’, while the absence of such findings was considered ‘4RT-like’. The classification results were then compared with the outcomes of the visual read. We also calculated the number of ROIs with *Z*-score > 1 in pre-central and parietal GM, cerebral WM and the basal ganglia presumably enriched with 4RT deposits for further defining the ‘4RT-like’ distribution.

Based on the combination of visual assessments and examination of SUVR *Z*-score heat maps, we classified the patients with bvFTD into ‘3RT-like’, ‘4RT-like’, ‘non-tau (tau-)’ and ‘Alzheimer’s disease–like’ subgroups. Cases with a discrepancy between the visual read and quantitative characterization were conceived as being ‘unclassified’.

Additionally, because tau depositions are not necessarily localized within the defined anatomical boundaries, we performed a voxel-based analysis of parametric ^18^F-florzolotau SUVR images acquired from each patient in comparison with the control group. We also performed an exploratory voxel-based analysis of ^18^F-florzolotau SUVR images to identify brain regions with increased ^18^F-florzolotau retentions in 3RT-like or 4RT-like bvFTD cases compared with the controls (for details, see Supplementary Methods 2).

### Statistical analysis

#### Correlations of ^18^F-florzolotau SUVRs by PVC with clinical measures in patients with tau-positive bvFTD

For patients with bvFTD who were judged as tau-positive by either visual read or quantitative assays of ^18^F-florzolotau-PET image assessments, we used the Spearman’s rank test to examine the correlation of radioligand retention with clinical and neuropsychological measures. ROIs determined by the aforementioned analysis were merged within the frontal, temporal and parietal GM areas and striatum. ^18^F-florzolotau SUVRs in these areas were estimated by PVC, and their correlations with total MMSE, FAB, NPI and SRI scores were statistically tested.

#### Associations of tau topologies with clinical characteristics and brain atrophy in patients with bvFTD

In cases with bvFTD, we used the Kruskal–Wallis test to investigate differences in clinical characteristics among tau topology subgroups categorized as 3RT-like, 4RT-like, tau-negative and Alzheimer’s disease–like. Test variables included the total scores of MMSE, FAB, NPI and SRI and 5 MMSE, 6 FAB, 12 NPI and 5 SRI subscales. Initial clinical symptoms and current neurological findings (presence or absence of parkinsonism) of the patients were also compared among the tau topology subgroups.

We also qualitatively assessed differences in the distribution of focal brain atrophy among the tau topology subgroups by using a *Z*-score map of regional brain volumes corrected for intracranial volume (ICV) as follows: *Z*-score = [(regional brain volume/ICV) in each subject−mean (regional brain volume/ICV) in healthy controls]/[standard deviation of (regional brain volume/ICV) in healthy controls].

The *Z*-score map was generated by assessing local atrophy in 64 ROIs used for the regional ^18^F-florzolotau SUVR assessments.

All statistical analyses were conducted with SPSS 23.0 (SPSS Inc.). The statistical significance threshold was set at *P* < 0.05 (two tailed).

## Results

### Demographic characteristics

Demographic data are shown in Table 1. There were no significant differences in age and sex between the patient and control groups. MMSE and FAB scores and educational levels were significantly lower in patients than in controls.

### Aβ positivity assessed by ^11^C-PiB-PET

Aβ depositions were observed by ^11^C-PiB in four patients with bvFTD and one with CBS. There were no control participants with Aβ positivity.

### Visual assessments of ^18^F-florzolotau SUVR images

**Figure 1:**
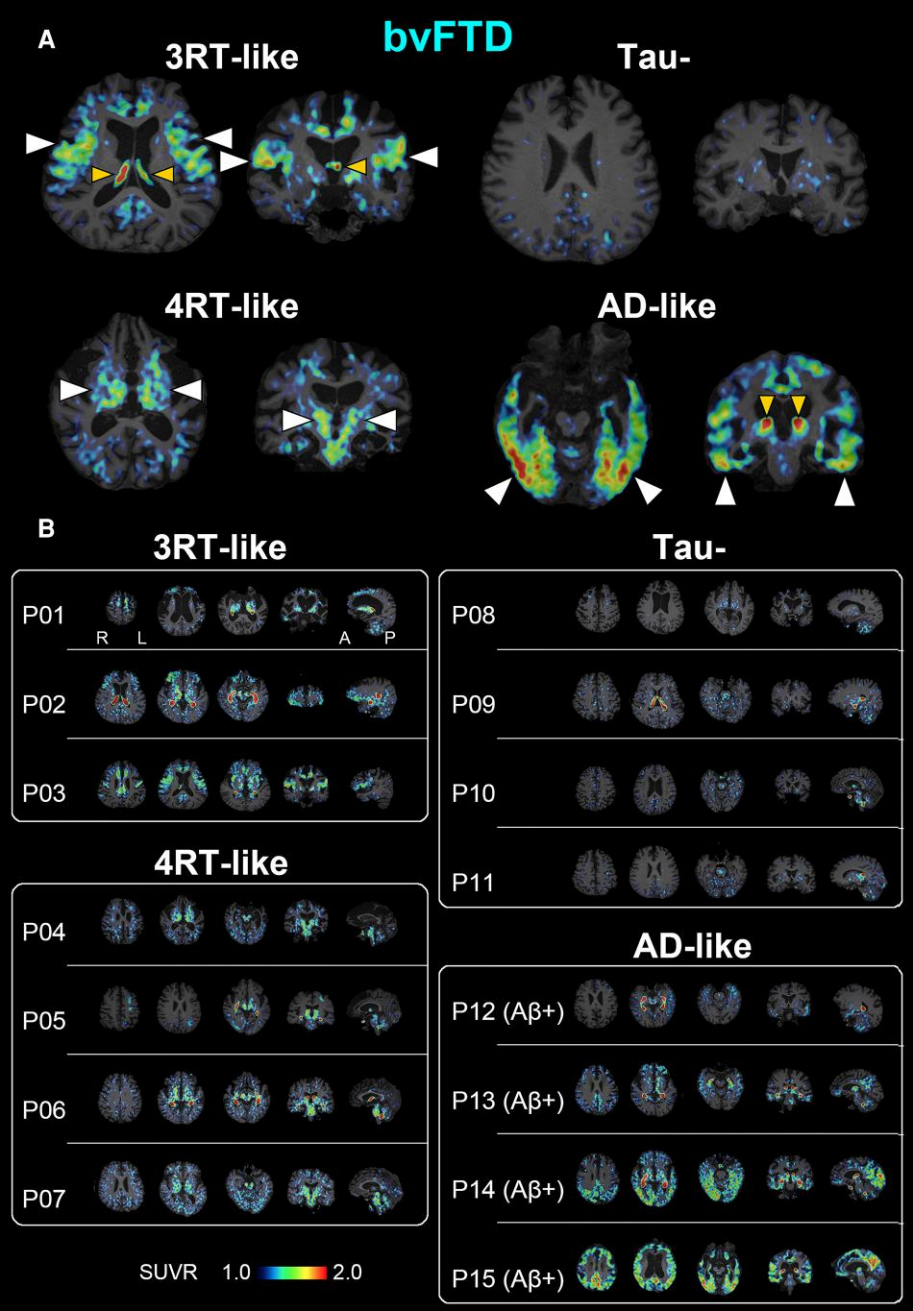
**A PET-based subtyping of bvFTD.** (**A**) Representative axial (left) and coronal (right) images of radioactivity SUVRs at 90–110 min after ^18^F-florzolotau administration. The arrowheads of normal size indicate enhanced parenchymal radioligand retentions characteristic of each putative tau topology subtype. The smaller arrowheads denote radioactivity accumulations in the choroid plexus supposedly unrelated to tau depositions. Radio signal intensification primarily in the frontal and temporal cortices in contrast to relative sparing of posterior and subcortical areas is indicative of Pick’s disease–type 3RT aggregations (P03), while involvements of subcortical regions, including the basal ganglia, thalamus, subthalamic nucleus and brainstem, suggest PSP- or CBD-type 4RT lesions (P04). The negativity for ^18^F-florzolotau-PET implies non-tau bvFTD exemplified by Type A TDP-43 pathologies (P11). A case with Aβ-PET–positive exhibited limbic and neocortical radioligand accumulations involving temporo-parietal regions and is accordingly granted a clinicopathological phenotype of bvFTD due to Alzheimer’s disease pathologies (P15). (**B**) A subgrouping of 15 cases with bvFTD by the visual read of individual tau PET images. Patients P01–P03 showed predominant ^18^F-florzolotau accumulations in the frontotemporal and frontolimbic cortices with minimal posterior cortical involvements suggestive of 3RT pathologies. P01 is a case with autopsy-confirmed Pick’s disease. P04–P07 presented increased radioligand retentions in subcortical structures accompanied by neocortical involvements to varying degrees, implying the presence of 4RT depositions. P08–P11 displayed no overt radioligand accumulations. P12–P15 were Aβ-positive, and the distribution of radio signals followed Braak tau staging consistent with Alzheimer’s disease. In each subject, three axial, one coronal and one sagittal sections providing characteristic information are presented from the left. A, anterior; L, left; P, posterior; R, right.

**Figure 2:**
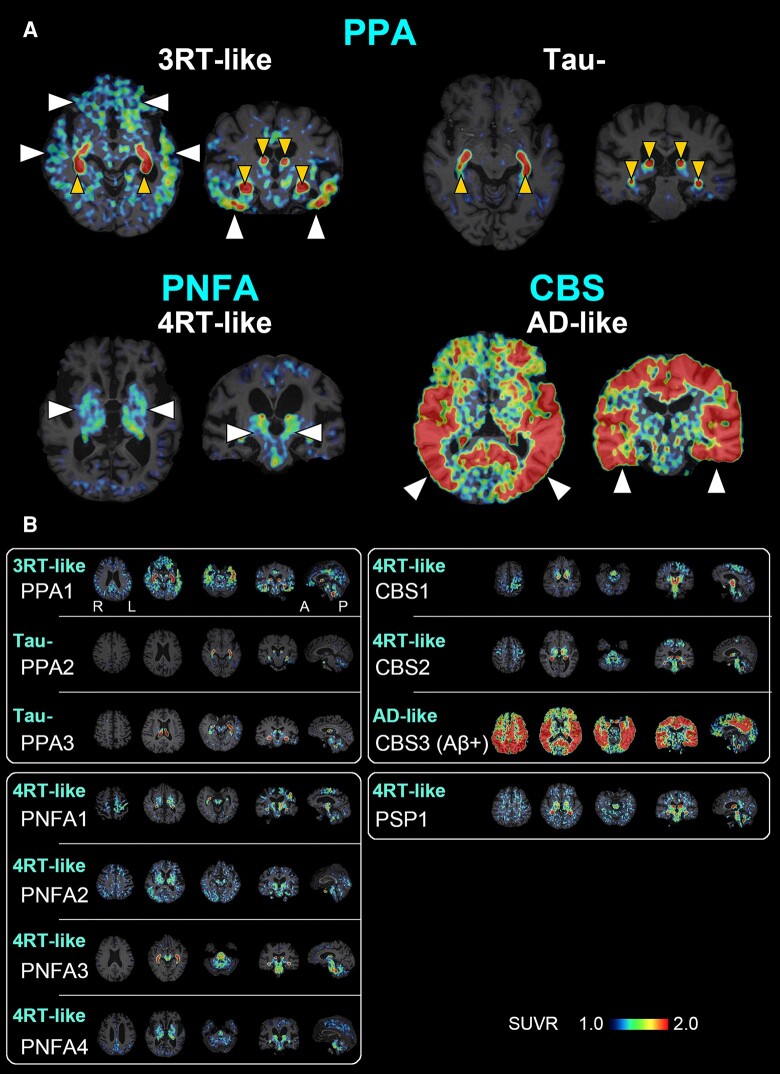
**A PET-based subtyping of PPA, PNFA, CBS and PSP.** (**A**) Representative axial (left) and coronal (right) images of radioactivity SUVRs at 90–110 min after ^18^F-florzolotau administration. The arrowheads of normal size indicate enhanced parenchymal radioligand retentions characteristic of each putative tau topology subtype. The smaller arrowheads denote radioactivity accumulations in the choroid plexus supposedly unrelated to tau depositions. Radio signal intensification primarily in the frontotemporal regions and less involvement of posterior and subcortical areas are suggestive of Pick’s disease–type 3RT aggregations in a case with PPA (PPA1), while negativity for tau depositions is indicated in another case with PPA (PPA2). Radio signal increases predominantly in subcortical regions implying PSP- or CBD-type 4RT deposits in a case with PNFA (PNFA4). A case with Aβ-PET–positive CBS (CBS3) exhibited widespread and highly intensified limbic and neocortical radioligand accumulations involving temporo-parietal regions, which is indicative of CBS due to Alzheimer’s disease pathologies. (**B**) The characterization of putative brain pathologies in three PPA (PPA1–3), four PNFA (PNFA1–4), three CBS (CBS1–3) and one PSP (PSP1) patients by the visual read of tau PET images. Cases with PPA were characterized as 3RT-like (PPA1) and tau-negative (PPA2 and 3), whereas all cases with PNFA were categorized as 4RT-like. Image findings in cases with CBS were either 4RT-like (CBS1, 2) or Alzheimer’s disease–like (CBS3), and radio signal distribution in the PSP-FTD case was consistent with 4RT-like pathology. In each subject, three axial, one coronal and one sagittal section providing characteristic information are presented from the left. A, anterior; L, left, P, posterior; R, right.

Individual ^18^F-florzolotau SUVR images of patients with bvFTD and with other FTD phenotypes are shown in Figs 1 and 2, respectively. By the visual read of these images, we classified the tau topologies of each subject as follows:

1. Patients with bvFTD

Among 15 cases with bvFTD, 7 individuals presented positivity for tau and negativity for Aβ, implying the presence of non–Alzheimer’s disease tau pathologies. Three of them (P01–P03) showed predominant ^18^F-florzolotau accumulations in the frontotemporal or frontolimbic cortices (upper left images in Fig. 1A; P01–P03 in Fig. 1B), the regions typically affected by 3RT pathology.^40^ The other four tau-positive and Aβ-negative patients showed less prominent ^18^F-florzolotau retentions in the frontal cortex and intensified radio signals in the temporo-parietal cortices, cerebral WM and subcortical structures, including subthalamic nucleus, striatum and midbrain (lower left images in Fig. 1A; P04–P07 in Fig. 1B), which were in line with regions affected by 4RT pathology.^42^ Four patients exhibited no overt increases of either ^18^F-florzolotau or ^11^C-PiB retentions in cortical and subcortical regions (upper right images in Fig. 1A; P08–P11 in Fig. 1B). Four patients with Aβ positivity showed accumulations of ^18^F-florzolotau radio signals in the neocortical and limbic cortices to varying degrees, with enhancements in the inferior and lateral temporal and posterior cingulate cortices, precuneus and temporo-parietal junction, which are characteristics of Alzheimer’s disease tau pathologies (lower right images in Fig. 1A; P12–P15 in Fig. 1B).

1. Patients with other FTD phenotypes

Similar to cases with bvFTD, 11 patients with FTD clinical phenotypes other than bvFTD were also classified into 4 categories according to tau topologies. All three patients with PPA were Aβ-negative, and one of them showed 3RT-like accumulations of ^18^F-florzolotau predominantly in the frontotemporal areas (upper left images in Fig. 1A; PPA1 in Fig. 2B). The other two patients with PPA did not present any unequivocal tau pathologies (upper right images in Fig. 2A; PPA2 and PPA3 in Fig. 2B).

All four patients with PNFA were Aβ-negative and presented 4RT-like tau topologies with predominance in the temporo-parietal cortices, cortical WM and subcortical areas (lower left in Fig. 2A; PNFA1–PNFA4 in Fig. 2B). Three cases with CBS consisted of two Aβ-negative individuals with 4RT-like tau depositions (CBS1 and CBS2 in Fig. 2B) and one Aβ-positive individual with Alzheimer’s disease–type tau distribution (lower right images in Fig. 2A; CBS3 in Fig. 2B). One patient with PSP-F was Aβ-negative and showed predominant ^18^F-florzolotau accumulations in the subthalamic nucleus, striatum and midbrain, suggesting the presence of 4RT lesions.

For inter-rater variability of the visual read, agreement among 3 readers across 21 images without Aβ positivity was high, with Fleiss’s *κ* of 0.75 (*P* < 0.001). Agreement between two judgements by the same reader was also high, with Cohen’s *κ* of 0.83–1.00 (*P* < 0.001 for all).

### Assessments of ^18^F-florzolotau accumulations based on SUVR *Z*-score maps

**Figure 3:**
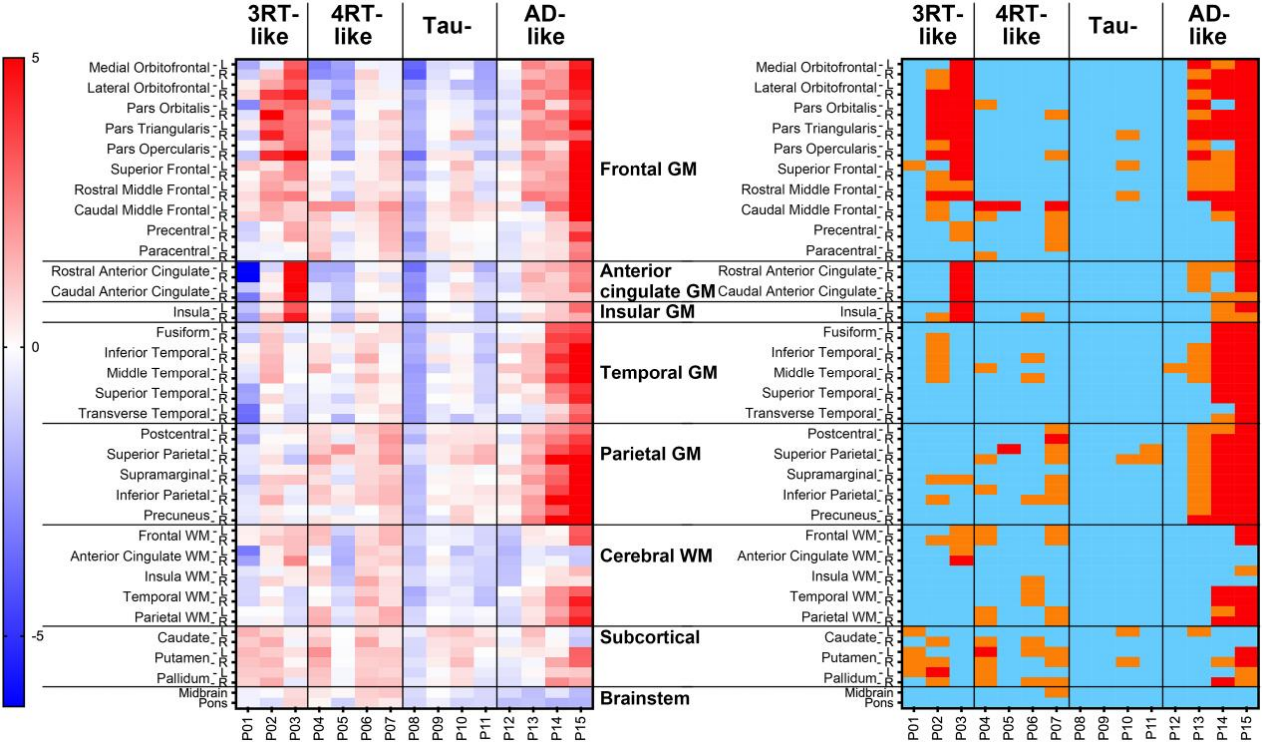
**Heatmaps of *Z*-scores for regional ^18^F-florzolotau SUVRs in patients with bvFTD (*n* = 15)**. Classification by visual read of tau PET images (see Fig. 1) is indicated on the top. The left panel displays uncategorized *Z*-scores. The right map presents the following *Z*-score ranges: light blue, <1; orange, 1–2; red, >2. P01–11 are Aβ-negative, and P12–15 are Aβ-positive.

In patients with bvFTD, tau depositions in the brains of cases with bvFTD were analysed by determining *Z*-scores for ^18^F-florzolotau retentions in 64 ROIs (Fig. 3). Among 11 patients with Aβ-negative bvFTD, 6 (P02–P07) were judged as tau-positive, as they possessed one or more ROIs with *Z*-score > 2 or 10 or more ROIs with *Z*-score > 1, in line with visual assessments. The patient with autopsy-confirmed Pick’s disease (P01) did not fulfil these criteria due to severe cortical atrophy and resultant partial volume effects. Four cases (P08–P11) were classified as consistently tau-negative by visual and *Z*-score–based examinations. In addition, SUVR *Z*-score maps of patients with other FTD phenotypes are shown in Supplementary Fig. 1.

**Figure 4:**
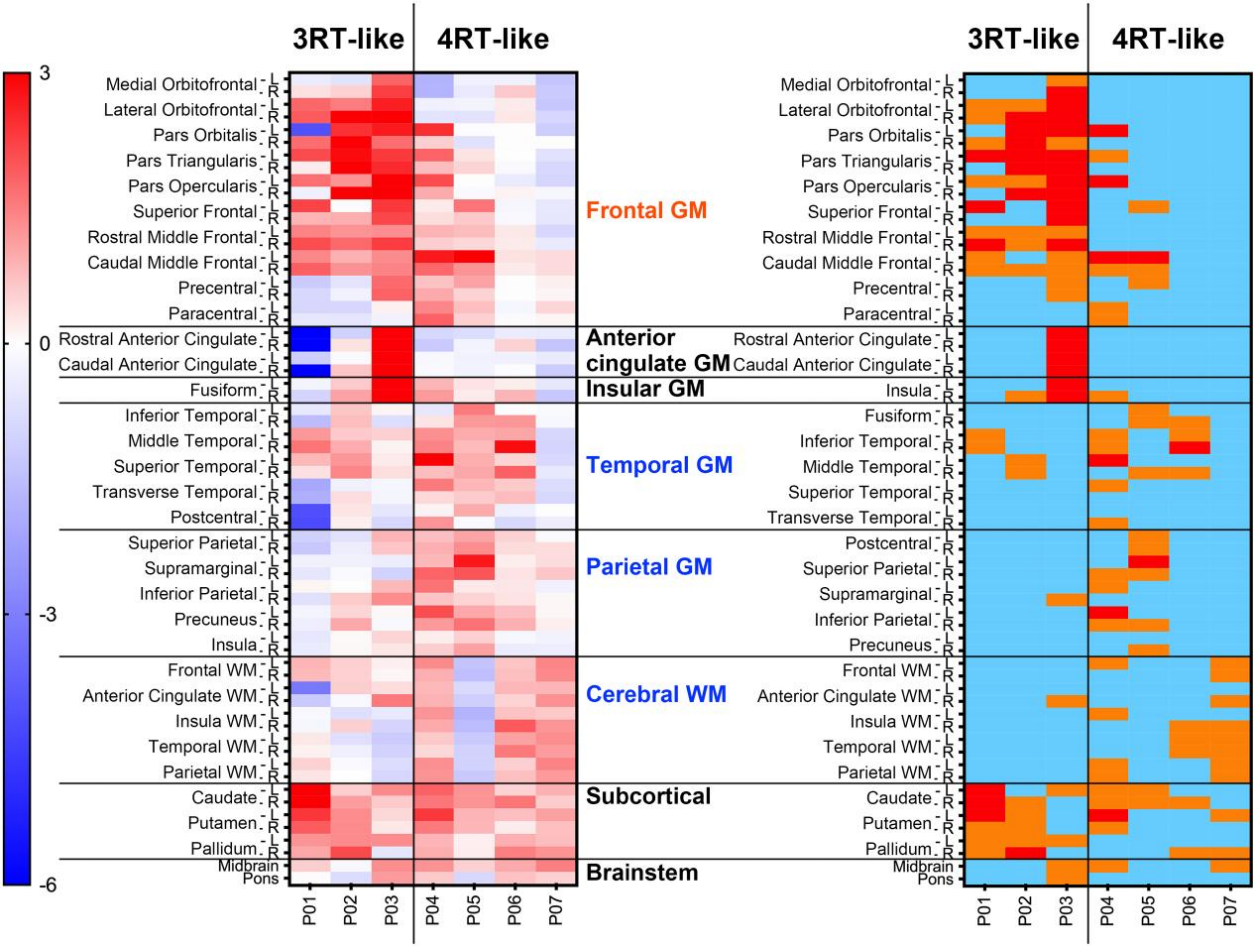
**Heatmaps of *Z*-scores for regional ^18^F-florzolotau SUVRs with PVC in patients with bvFTD with 3RT- and 4RT-like radio signal accumulations by visual read (*n* = 7).** Classification by visual read of tau PET images (see Fig. 1) is indicated on the top. The left panel displays uncategorized *Z*-scores. The right map presents the following *Z*-score ranges: light blue, <1; orange, 1–2; red, >2. Patients P01–P03, who were indicated as possessing 3RT-like pathologies by visual inspections of images, had 10 or more ROIs with *Z*-score > 1 within the inferior and middle pre-frontal GM, the inferior, middle and superior temporal GM and the caudate. In contrast, the other four patients (P04–P07) who were visually estimated to possess 4RT-like tau pathologies did not meet the above *Z*-score criterion for 3RT and had multiple ROIs with *Z*-score > 1 within pre-central and parietal GM, cerebral WM and the basal ganglia.

We then applied PVC to ^18^F-florzolotau SUVR images of seven Aβ-negative bvFTD patients with tau positivity in the visual read (P01–P07) and calculated the *Z*-score in each ROI (Fig. 4). In agreement with visual subtyping, tau topologies of 3 cases (P01–P03) were considered to be 3RT-like because they had 10 or more ROIs with *Z*-score > 1 within the inferior and middle pre-frontal GM, the inferior, middle and superior temporal GM and the caudate (right panel in Fig. 4). In contrast, the other four patients (P04–P07) who were visually estimated to possess 4RT-like tau pathologies did not meet the *Z*-score criterion for 3RT. Notably, all these patients presented high *Z*-scores in multiple ROIs within pre-central and parietal GM, cerebral WM and the basal ganglia (right panel in Fig. 4). We accordingly defined an arbitrary criterion for 4RT-like tau topology as five or more ROIs with *Z*-score > 1 in pre-central and parietal GM, cerebral WM and basal ganglia, in addition to the lack of meeting the above criterion for 3RT-like tau pathologies.

Results of our voxel-based comparisons of each patient’s SUVR images with the controls generally agreed with our visual read and ROI-based quantitative assessments (for details, see Supplementary Results 1 and Fig. 2). Results of our exploratory voxel-based comparisons between the cases and controls with 3RT-like bvFTD and between the cases and controls with 4RT-like bvFTD were in agreement with the topologies of 3RT and 4RT pathologies in previous post-mortem examinations^2,40^ (for details, see Supplementary Results 1 and Fig. 3).

### Associations of tau PET findings with clinical characteristics and brain atrophy in bvFTD patients

**Figure 5:**
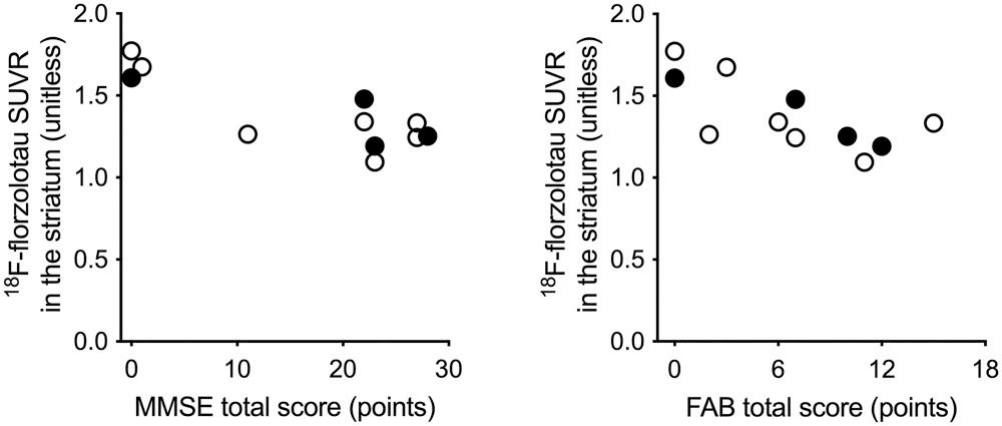
**Scatter plots of ^18^F-florzolotau SUVRs with PVC in the striatum against total scores of MMSE and FAB in patients with tau-positive bvFTD (total *n* = 11).** Filled circles indicate patients with Aβ positivity assessed by ^11^C-PiB (*n* = 4).

We estimated ^18^F-florzolotau SUVRs with PVC in the merged frontal, temporal and parietal GM and striatal ROIs and assessed their correlations with MMSE, FAB, NPI and SRI scores. SUVRs in the striatum were significantly and negatively correlated with total scores of MMSE (rho = −0.75, *P* = 0.008) and FAB (rho = −0.71, *P* = 0.01; Fig. 5), but no other correlations were found to be statistically significant (*P*-value range, 0.21–0.96). In our supplementary voxel-based analysis of parametric ^18^F-florzolotau SUVR images with MR-based PVC, no significant correlations between SUVR and total MMSE or FAB scores were found within the cortical GM regions mentioned above (for details of the methods, see Supplementary Methods 3).

**Figure 6:**
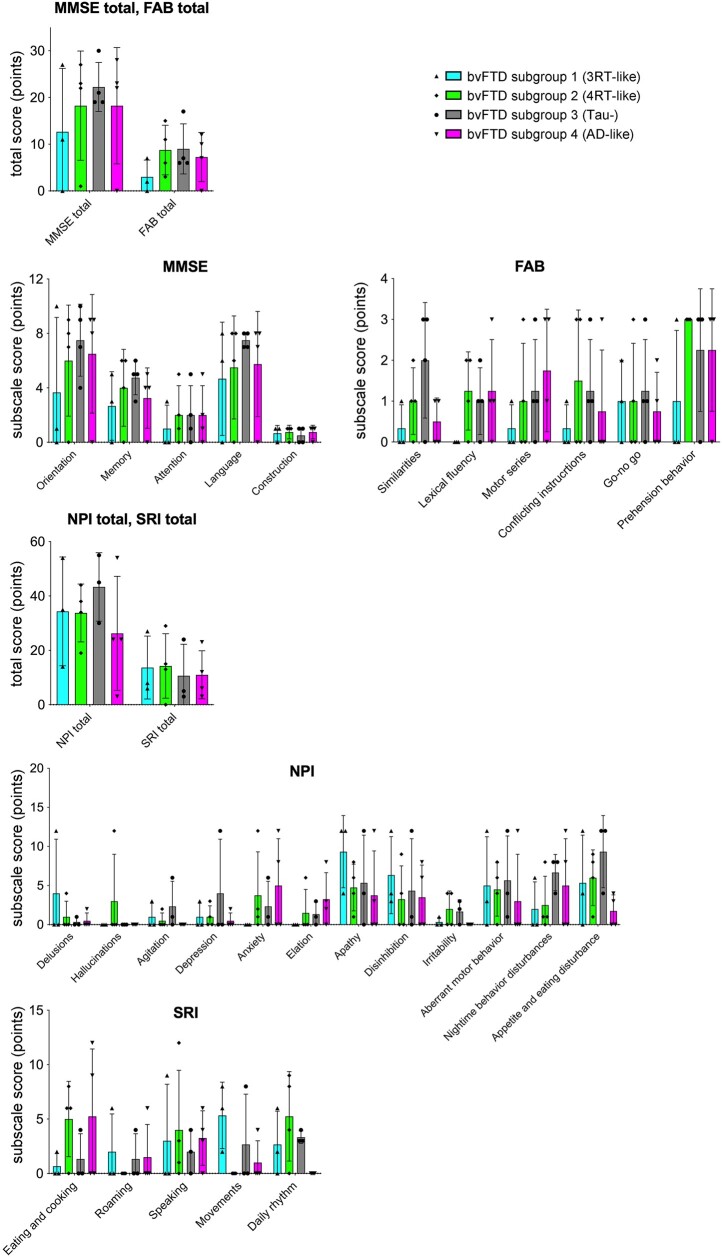
**Comparisons of clinical measures among PET-based neuropathological subgroups of bvFTD.** By visual read and *Z*-score mapping of tau PET data, cases with bvFTD were classified into ‘3RT-like’ (Subgroup 1; *n* = 3), ‘4RT-like’ (Subgroup 2; *n* = 4), ‘tau-’ (Subgroup 3; *n* = 4) and ‘Alzheimer’s disease–like’ (Subgroup 4; *n* = 4) subgroups. Lower scores of MMSE and FAB indicate worse cognition or function, while higher scores of NPI and SRI indicate more severe symptoms. There were no significant subgroup differences in these clinical variables by the Kruskal–Wallis test. Solid symbols denote individual values. NPI and SRI data for one patient in Subgroup 3 are not available.

The Kruskal–Wallis test showed no significant differences in the total scores of MMSE, FAB, NPI or SRI among four tau topology subgroups in patients with bvFTD (*P*-value range, 0.50–0.86). Although there were trends towards differences in the SRI subscale scores of ‘movements’ (*P* = 0.08) and ‘daily rhythm’ (*P* = 0.09), no significant differences were found in the subscale scores of MMSE (*P*-value range, 0.15–0.97), FAB (*P*-value range, 0.23–0.93), NPI (*P*-value range, 0.51–0.87) or SRI (*P*-value range, 0.08–0.90) among the tau topology subgroups (Fig. 6). The initial clinical symptoms and the presence or absence of current parkinsonism in patients in each subgroup are summarized in Supplementary Table 1. Disinhibitions were observed only in cases with 3RT-like tau distribution, and depression was a frequent initial manifestation in tau-negative cases. Apart from these tendencies, no marked subgroup-specific symptomatic distinctions at clinical onset were noted. Parkinsonism was present at the time of PET examinations in none or only one case in each subgroup.

**Figure 7:**
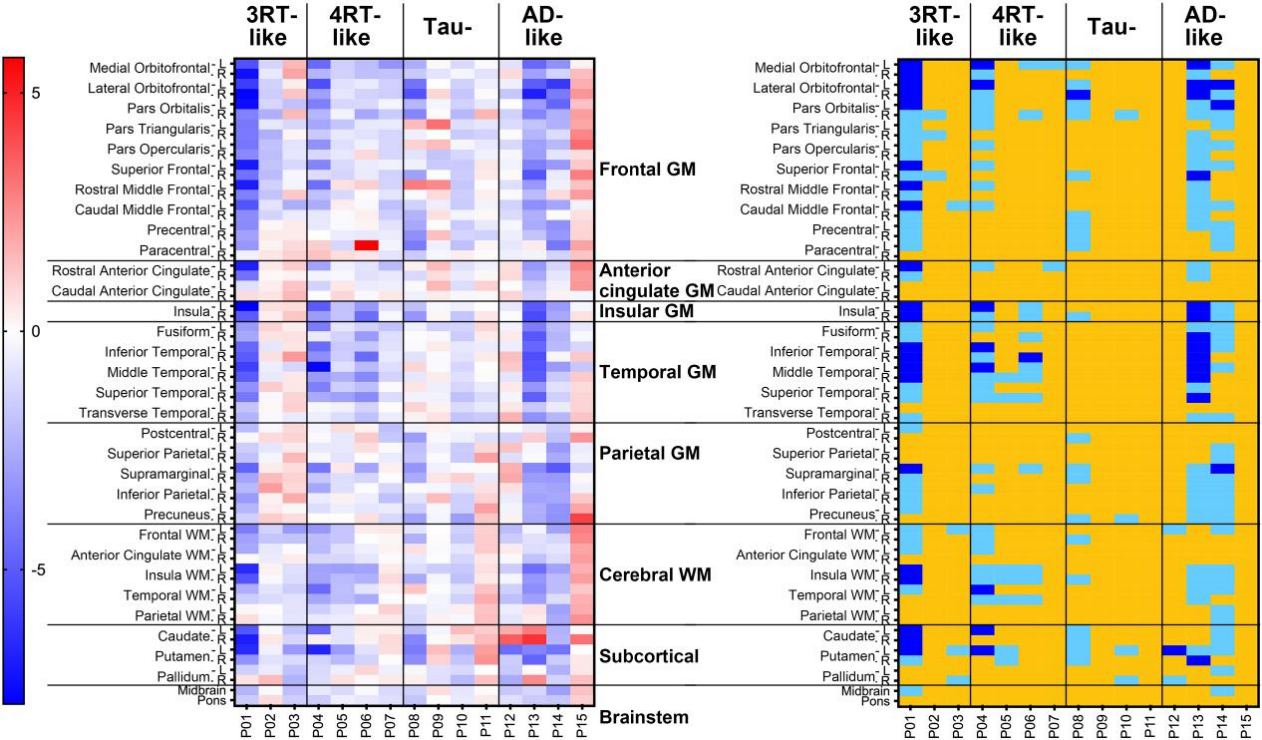
**Heatmaps of *Z*-scores for regional volumes divided by ICV in patients with bvFTD.** Classification by visual read of tau PET images (see Fig. 1) is indicated on the top. The left panel displays uncategorized *Z*-scores. The right map presents the following *Z*-score ranges: blue, <−4.5; light blue, −4.5 to −2; yellow, >−2. There are no overt associations between the regionality of the volume loss and putative neuropathological phenotypes in these subjects.

As shown in Fig. 7, brain atrophy in cases with bvFTD showed sizeable individual variability but no regional characteristics of each tau topology subgroup. In principle, the temporal and parietal GM and cerebral WM were relatively spared in subjects with putative 3RT depositions but were notably affected in cases with putative 4RT depositions. However, such features were prominently masked by extensive volume reductions seemingly associated with disease durations, as exemplified by P01 with autopsy-defined Pick’s disease.

## Discussion

The current experimental data demonstrated the potential capability of PET scans for estimating neuropathological subtypes of FTD according to tau topologies. By taking advantage of ^18^F-florzolotau in the high-contrast detection of Alzheimer’s disease and non–Alzheimer’s disease tau assemblies with distinct isoform compositions,^17^ the regionality of tau pathologies in cases with FTD was classified into 3RT-like, 4RT-like, tau-negative and Alzheimer’s disease–like subcategories. The visual read and *Z*-score mapping of tau PET images led to consistent subgrouping of patients with bvFTD, while clinical and MRI volumetric profiles did not remarkably differ among the tau topology subtypes. These observations indicate that neuropathological phenotypes underlying this clinical syndrome cannot be estimated by either symptomatic features or localizations of atrophy, highlighting the role of ^18^F-florzolotau-PET in the biological stratification of individuals with FTD.

Recent cryo-electron microscopic analysis of tau fibrils extracted from tauopathy cases has revealed that specific ultrastructures of tau filaments are primary determinants of subcellular, cellular and regional localization of tau lesions in each illness.^43^ The extent of neuronal tau depositions in Alzheimer’s disease is largely affected by Aβ pathologies and follows Braak staging with the advancement of the disease.^17,35^ At early and middle clinical stages, neuronal 3RT deposits are rather confined to the frontal and temporal cortical GM in Pick’s disease, while temporo-parietal GM, widespread cortical WM and subcortical structures are also involved in neuronal, astrocytic and oligodendrocytic 4RT pathologies in PSP and CBD.^17,44^ It is noteworthy that PET with ^18^F-florzolotau is able to detect tau aggregates in the posterior cortical regions but may not be highly sensitive to frontal tau inclusions in PSP.^17,20,45^ Accordingly, frontolimbic and frontotemporal predominance of ^18^F-florzolotau retentions could be an indicator for 3RT depositions, and intensifications of tau probe radio signals in parietotemporal versus frontotemporal GM, along with cortical WM, might suggest 4RT accumulations.

Our results have illustrated consistency between classifications of cases with bvFTD by visual inspection and region-wise statistical examination of tau PET images, whereas objective criteria for categorizing putative pathological entities of this clinical syndrome would need to be carefully defined. Most patients with 4RT-like tau topology present visually noticeable enhancements of ^18^F-florzolotau retentions in subcortical areas, including the basal ganglia, subthalamic nucleus, brainstem and deep cerebellar structures. However, *Z*-scores in these regions of 4RT-like bvFTD patients did not markedly differ from those of other cases. This dissociation might stem from a lower abundance of subcortical tau deposits in FTD individuals with equivocal motor impairments than in PSP patients with disordered movements. As a technical consideration, previous studies have indicated lower non-specific binding of ^18^F-florzolotau in the basal ganglia than preceding radioligands such as ^11^C-PBB3. An *in vitro* binding assay has shown minimal displacement of ^18^F-florzolotau with a monoamine oxidase-A inhibitor, clorgiline, or a monoamine oxidase-B inhibitor, selegiline, suggesting that it barely cross-reacts with off-target binding sites on these enzymes.^17^ However, as we cannot exclude the possibility that ^18^F-florzolotau might react with other unidentified off-target binding components in the basal ganglia, interpretation of its radio signals in this area should be conducted with caution. Regarding the cortical distribution of 4RT pathologies, PSP and CBD generally show symmetric and asymmetric tau accumulations, respectively. However, asymmetry of the PET tracer retentions was not apparent in the majority of cases with bvFTD in this study. The patients with bvFTD in our study did not present motor symptoms, and it is likely that tau depositions in the pre-central cortex, basal ganglia and associated areas of these individuals are less abundant than those of cases with motor phenotypes, hampering the differentiation between PSP and CBD. Following the PVC of ^18^F-florzolotau SUVR images, we demonstrated that the presence of 10 or more ROIs with *Z*-score > 1 within the inferior and middle pre-frontal GM, the inferior, middle and superior temporal GM, and the caudate could be a criterion for 3RT-like distribution of tau pathologies, while 4RT-like radio signal intensification was suggested by the existence of 5 or more ROIs with *Z*-score > 1 in the pre-central and parietal GM, cerebral WM,- and the basal ganglia, in addition to the lack of meeting the above criterion for 3RT-like tau pathologies.

It is of note that these categorizations were more valid in cases at the early and middle stages of the disease than in those with advanced neurodegenerative alterations. Indeed, a severely affected patient with bvFTD (P01) presented prominent atrophy of widespread cortical structures, hampering the detection of tau accumulations as significant elevations of *Z*-scores (Fig. 3). We observed multiple ROIs with *Z*-score > 1 in the frontal GM of this case following PVC (Fig. 4), yet the corrected SUVRs may not sufficiently reflect high loads of 3RTs confirmed as numerous Pick bodies in the post-mortem analysis.^17^ This discrepancy should not be an issue in an early diagnostic assessment of individuals with FTD in coordination with emerging anti-tau treatments, whereas the PVC methodology may need to be optimized for examining imaging–neuropathology correlations in the same individual with marked neuronal loss.

The categorization of tau topologies demonstrated in patients with bvFTD appeared to be valid in other FTD syndromes, although *Z*-map–based classification with assigned criteria was not conducted due to the insufficient number of subjects with each clinical phenotype. One of the three cases with PPA presented 3RT-like tau depositions, which agreed with a past neuropathological finding that 3RT pathology was frequently present in patients with PPA.^4^ The other two subjects with PPA were negative for the tau PET examination, which may be consistent with the neuropathological evidence that TDP-43 is deposited in more than half of cases with PPA.^4^ The implication of TDP-43 pathologies is also likely in patients with tau-negative bvFTD,^4,46^ and neuroimaging- and fluid-based biomarkers for TDP-43 aggregations in the brain are required for confirming this possibility. It is noteworthy that all four cases with PNFA presented 4RT-like tau depositions according to visual reads, in concordance with the high frequency of CBD and PSP pathologies in this clinical FTD subtype.^4^ Moreover, 4RT deposits were indicated in two of three subjects with CBS and in one subject with FTD-PSP clinical phenotype. The radio signal distribution in these subjects was similar to the previous tau PET findings in patients with PSP and putative CBD,^20,37,38,47^ whereas the distinction between PSP and CBD tau topologies was not so distinct. Besides primary tauopathies and non-tau proteinopathies, enhanced tau PET radio signals were acquired in all patients with Aβ-positive —in four subjects with bvFTD and one subject with CBS. The extent of tau lesions in these individuals followed Braak tau stages with minimal subcortical involvements, suggesting that Alzheimer’s disease rather than 4RT or 3RT pathologies underlay FTD manifestations.

To investigate the compositions of probable neuropathological subtypes in patients with bvFTD, it should be taken into account that clinicians’ diagnostic confidence could influence the frequency of certain pathological entities.^46^ We performed an auxiliary analysis to address this issue (procedures are described in Supplementary Methods 4). Our results showed that the proportion of Alzheimer’s disease–like bvFTD was notably small (only one case) in the ‘high diagnostic confidence’ group (Supplementary Results 2 and Fig. 4), in line with a previous clinicopathological study.^46^ In addition, the proportions of 3RT-like, 4RT-like, non-tau and Alzheimer’s disease–like cases in the ‘high diagnostic confidence’ group resembled those in the previous report.^46^ Given that a significant portion of cases with tau-negative bvFTD possessed TDP-43 inclusions, the high frequency of tau PET negativity in the ‘high diagnostic confidence’ group relative to the total bvFTD population in the present work is also in accordance with the previous data.^46^

Our clinical assessments showed that patients with bvFTD in this study had severe apathy, disinhibition, behavioural disturbances and diet- and sleep-related disturbances (Fig. 6), which were all generally in agreement with the known symptomatic features of this FTD subcategory.^23^ In addition, tau accumulations in the striatum were inversely correlated with cognitive abilities and frontal functions as assessed by MMSE and FAB, respectively (Fig. 5). These results align with a previous observation that striatal dysfunction contributed to alterations in cognition and behaviours in neurodegenerative conditions.^48^ Tau depositions in the limbic and neocortical regions were diverse in line with the topological subgroups, such as 3RT-like and 4RT-like distributions, and were not closely associated with MMSE and FAB scores.

Among the four tau topology subgroups, there were no marked differences in the clinical and symptomatic severity assessed by MMSE, FAB, NPI and SRI, the characteristics of initial symptoms, or the frequency of parkinsonism. Likewise, a previous study of clinicopathological correlations reported that frequencies of the core clinical features did not overtly differ among bvFTDs due to tau, TDP-43 and FUS pathologies.^46^ In addition, the current work has not demonstrated any clear distinctions of focal brain atrophy among the four tau topological subgroups. This finding is also consistent with the similarity of the localization of morphometric changes among cases with bvFTD with 3RT, 4RT and TDP-43 pathologies or among their subclassifications.^46,49^ Collectively, these results indicate that the molecular pathologies in patients with bvFTD are barely predictable from symptomatic and MRI volumetric features and that they need to be more directly assayed with specific imaging agents.

There are several technical limitations in the present study. First, the sample size was relatively small. Second, this study used a cross-sectional design, and we were not able to examine trajectories of tau accumulations along the course of the illness in each patient. Although we confirmed that there were no changes in the clinical diagnosis of any of the patients with FTD in our study for at least 1 year after the PET scan, longitudinal PET data, in addition to detailed clinical follow-up information, are needed to further clarify the relationship among the clinical features of FTD- and PET-assessed tau pathologies during the course of the illness. Third, the possible influence of the ROI definition and regional brain atrophy on the results of PET analysis may need to be tested in a larger group of subjects. Finally, although one patient with autopsy-confirmed Pick’s disease was included in this study, no other neuropathological examinations were performed in our dataset. Future studies of imaging–pathology correlation using ^18^F-florzolotau in patients with FTD due to diverse tau pathologies, including Pick’s disease, CBD and PSP, will be required to validate the capability of this radioligand for detecting and differentiating various FTLD tauopathies *in vivo*.

## Conclusion

The present PET study of cases with FTD provides evidence that heterogeneous tau pathologies derived from distinct isoform compositions could be identified in each subject with the aid of ^18^F-florzolotau. Our data also indicate the complexity of tau–symptom relationships, possibly mediated by neural connections between the tau lesion and the core region responsible for clinical manifestations. The stratification of FTD individuals according to putative tau pathology subtypes will represent an essential utility for emerging treatments with antibodies and antisense oligonucleotides targeting tau species,^50,51^ which would be more specific for either 3RT or 4RT. The mechanistic links of PET-visible tau deposits to brain atrophy and clinical deterioration will also need to be pursued along with such therapeutic interventions.

## Supplementary Material

fcae075_Supplementary_Data

## Data Availability

The datasets generated and analysed during the current study are available from the corresponding author upon reasonable request.
