# Supplementary material for: In vivo PET classification of tau pathologies in patients with frontotemporal dementia
Source: Brain Commun. 2024 Mar 1;6(2):fcae075. doi: 10.1093/braincomms/fcae075 (PMC10953627; doi:10.1093/braincomms/fcae075)
Supplement: fcae075_Supplementary_Data [file fcae075_supplementary_data.pdf]

SUPPLEMENTARY MATERIAL

Title: ***In vivo*** **PET classification of tau pathologies in patients with frontotemporal dementia**

Authors:

Manabu Kubota,[1,2] Hironobu Endo,[1] Keisuke Takahata,[1,3] Kenji Tagai,[1,4] Hisaomi Suzuki,[1,5] Mitsumoto Onaya,[5] Yasunori Sano,[1,3] Yasuharu Yamamoto,[1,3] Shin Kurose,[1,3] Kiwamu Matsuoka,[1,6] Chie Seki,[1] Hitoshi Shinotoh,[1] Kazunori Kawamura,[7] Ming-Rong Zhang,[7] Yuhei Takado,[1] Hitoshi Shimada,[1,8] Makoto Higuchi[1]

1 Department of Functional Brain Imaging, Institute for Quantum Medical Science, Quantum Life and Medical Science Directorate, National Institutes for Quantum Science and Technology, 4-9-1 Anagawa, Inage, Chiba, Chiba 263-8555, Japan

2 Department of Psychiatry, Kyoto University Graduate School of Medicine, 54 Shogoin Kawahara-cho, Sakyo-ku Kyoto 606-8507, Japan

3 Department of Neuropsychiatry, Keio University School of Medicine, 35 Shinanomachi, Shinjuku, Tokyo 160-8582, Japan

4 Department of Psychiatry, Jikei University Graduate School of Medicine, Tokyo 105-8461, Japan

5 Shimofusa Psychiatric Center, Chiba, 578 Heta-cho, Midori-ku, Chiba 266-0007, Japan

6 Department of Psychiatry, Nara Medical University, 840 Shijo-cho, Kashihara, Nara 634-8521, Japan

7 Department of Advanced Nuclear Medicine Sciences, Institute for Quantum Medical Science, Quantum Life and Medical Science Directorate, National Institutes for Quantum Science and Technology, 4-9-1 Anagawa, Inage, Chiba, Chiba 263-8555, Japan

8 Department of Functional Neurology & Neurosurgery, Center for Integrated Human Brain Science, Brain Research Institute, Niigata University, 1-757 Asahimachi-Dori, Chuo, Niigata, Niigata 951-8585, Japan

Corresponding author:

Manabu Kubota M.D., Ph.D.

Department of Functional Brain Imaging, Institute for Quantum Medical Science, Quantum Life and Medical Science Directorate, National Institutes for Quantum Science and Technology

Address: 4-9-1 Anagawa, Inage-ku, Chiba, Chiba 263-8555, Japan

Tel. +81-43-206-3251 Fax. +81-43-253-0396

Email: kubota.manabu@qst.go.jp; m_kubota@kuhp.kyoto-u.ac.jp

Table of contents

Supplementary Methods 1
Details of PET acquisition

Supplementary Methods 2
Voxel-based comparison of ^{18}F-florzolotau standardized uptake value ratio (SUVR) images

Supplementary Methods 3
Voxel-based correlational analysis of ^{18}F-florzolotau SUVR with MMSE and FAB scores in tau-positive bvFTD patients

Supplementary Methods 4
Associations between diagnostic confidence and tau topology subgroups in patients with bvFTD

Supplementary Results 1
Voxel-based comparison of ^{18}F-florzolotau SUVR images

Supplementary Results 2
Associations between diagnostic confidence and tau topology subgroups in patients with bvFTD

Supplementary Figure 1
Heatmaps of Z-scores for regional ^{18}F-florzolotau SUVRs in patients with non-behavioral-variant FTD phenotypes (n = 15)

Supplementary Figure 2
Results of voxel-based analysis on an individual patient level showing regions with higher ^{18}F-florzolotau SUVR as compared to the controls

Supplementary Figure 3
Voxel-wise group comparisons to identify regions with significantly higher SUVR in 3RT-like (P01-P03) and 4RT-like (P04-P07) bvFTD cases than 20 controls

Supplementary Figure 4
Tau pathology classifications of bvFTD cases based on ^{18}F-florzolotau-PET findings for (a) all participants and (b) those clinically diagnosed with high certainty

Supplementary Table 1
Initial clinical symptoms and current neurological findings (presence or absence of parkinsonism) of patients in each bvFTD subgroup classified by our ^{18}F- florzolotau assessments

Supplementary References

Supplementary Methods

Supplementary Methods 1

Details of PET acquisition

1) ^{11}C-PiB PET

50 min after an intravenous rapid bolus injection of ^{11}C-PiB, a 20-min PET acquisition (4×5-min frames) was performed with either a Biograph mCT flow system (Siemens Healthcare, Erlangen, Germany) (patients, n = 20; controls, n = 15) or with a Siemens ECAT Exact HR+ system (CTI/Siemens, Knoxville, TN, USA) (patients, n = six; controls, n = five).

For PET scans with Biograph mCT, the mCT flow system provided 109 sections with an axial field-of-view (FOV) of 21.8 cm. The intrinsic spatial resolution was 5.9 mm in-plane and 5.5 mm full-width at half-maximum axially. Images were reconstructed using a filtered back projection algorithm with a Hanning filter (4.0 mm full-width at half-maximum). Attenuation correction was applied based on computed tomography images, and correction for randoms was performed using the late coincidence counting method.

For PET scans with an ECAT Exact HR+, the HR+ system provided 63 sections with an axial FOV of 15.5 cm, and contiguous 2.46-mm slices with 5.6-mm transaxial and 5.4-mm axial resolution. Images were reconstructed by a filtered back-projection method (Hanning filter, cutoff frequency: 0.4 cycle/pixel). Attenuation correction was applied for each image using 10-min transmission scan data with a ^{68}Ge-^{68}Ga line source.

2) ^{18}F-florzolotau PET

90 min after an intravenous rapid bolus injection of ^{18}F-florzolotau in a dim room to avoid photo racemization, a 20-min PET acquisition (4×5-min frames) was performed with the Biograph mCT flow system (see ^{11}C-PiB PET section for scan parameters).

Supplementary Methods 2

Voxel-based comparison of ^{18}F-florzolotau standardized uptake value ratio (SUVR) images

We performed voxel-based analysis of parametric ^{18}F-florzolotau SUVR images of each patient in comparison with the control group using Statistical Parametric Mapping software (SPM12; Wellcome Department of Imaging Neuroscience, London, UK). For image preprocessing, MRI data of each participant were processed using SPM12 software, specifically using the Diffeomorphic Anatomical Registration Through Exponentiated Lie algebra (DARTEL) toolbox running on Matlab R2020b (MathWorks, MA). The segmented MRI and ^{18}F-florzolotau SUVR images of all participants were then spatially normalized into the standardized Montreal Neurological Institute (MNI) 152 brain template. The normalized SUVR images were smoothed with a Gaussian kernel of 6 mm full-width at half-maximum.

To investigate cortical regions in which a patient with frontotemporal dementia (FTD) shows higher ^{18}F-florzolotau retentions, two sample t-tests were performed in SPM12 to compare the SUVR images of each patient with the 20 healthy participants, controlling for total brain volume (TBV). Analysis was restricted to gray matter (GM) and white matter (WM) of the frontal, temporal, and parietal cortices (a mask was created by merging corresponding FreeSurfer's ROIs and by normalizing them into MNI space). The occipital cortex was excluded from the analysis since it is not critically involved in tau pathologies of FTD. Because of the exploratory nature of this study, we initially applied a lenient voxel-level height threshold of $p < 0.001$, uncorrected, with an extent threshold of 20 voxels, to supplement our quantitative assessments of cortical tau positivity. We subsequently applied a cluster-level family-wise error (FWE) correction of $pFWE < 0.05$.

We also performed an exploratory voxel-based analysis of ^{18}F-florzolotau SUVR images to identify brain regions with enhanced radiotracer retentions in either three-repeat tau isoform (3RT)-like or four-repeat tau isoform (4RT)-like behavioral variant FTD (bvFTD) patients compared to the controls. Two sample t-tests were performed in SPM12, and the same statistical thresholds and the cortical mask, as mentioned above, were used.

Supplementary Methods 3

Voxel-based analysis of correlations of ^{18}F-florzolotau SUVR with MMSE and FAB scores in tau-positive bvFTD patients

To explore brain areas with associations between cortical tau accumulations and total scores of MMSE and FAB in bvFTD patients who were judged as tau-positive by our ^{18}F-florzolotau assessments, we performed voxel-based correlational analysis using SPM12. MR-based partial volume correction (PVC) was applied to ^{18}F-florzolotau SUVR images using the Müller-Gärtner approach.[1] SUVR images of these patients with PVC were spatially normalized into the MNI 152 brain template and were smoothed with a Gaussian kernel of 6 mm full-width at half-maximum.

We applied multiple regressions in SPM12 to explore cortical regions in which SUVR values positively or negatively correlated with the MMSE or FAB scores, controlling for TBV. In accordance with our ROI-based correlational analysis, the voxel-based analysis was restricted to frontal, temporal, and parietal GM (mask was created by merging corresponding FreeSurfer's ROIs and normalizing them into MNI space). The voxel-based correlations of SUVRs with MMSE or FAB scores were investigated in separate models in SPM12. Because positive and negative correlations were examined in two different contrasts in SPM12, we set a voxel-level height statistical threshold of $p < 0.0005$, uncorrected, with an extent threshold of 20 voxels.

Supplementary Methods 4

Associations between diagnostic confidence and tau topology subgroups in patients with bvFTD

We investigated whether the proportion of each putative neuropathological background varies with diagnostic certainty in patients with bvFTD. For this purpose, we divided patients with bvFTD into either high, intermediate, or low diagnostic confidence based on the following criteria, similar to a previous study of clinicopathological correlations[2]: a) "high diagnostic confidence", bvFTD as a primary diagnosis at any visit to either a board-certified psychiatrist or a board-certified neurologist; b) "intermediate diagnostic confidence", diagnosis varied over time; or c) "low diagnostic confidence", diagnosis of bvFTD listed as an additional syndrome.

We compared the proportions of the tau topology subgroups classified by our ^{18}F-florzolotau assessments between the total sample of bvFTD and the high diagnostic confidence of bvFTD. Because of the small sample size in our study, we did not perform comparisons among the high, intermediate, and low confidence groups of bvFTD.

Supplementary Results

Supplementary Results 1

Voxel-based comparison of ^{18}F-florzolotau SUVR images

1) Patients with bvFTD

Compared to controls, seven Aβ-negative patients categorized as tau-positive by our visual read of ^{18}F-florzolotau images (P01-P07), and three out of four Aβ-positive patients (P13-P15) displayed regions with significantly higher SUVR values with voxel-level height threshold of $p < 0.001$, uncorrected, and extent of > 20 voxels (Supplementary Figure 2). These regions were generally in line with our visual read and ROI-based assessments by the SUVR Z-score map. In patients P01, P02, and P03, significantly higher SUVR values were found mainly in regions within the inferior frontal cortex, some of which extended into the middle frontal cortex (P01 and P02) or anterior cingulate and insula (P03). In contrast, patients P04-P07 showed significantly higher SUVR in the parietal GM (P04, P05, P07) or WM (P04, P06) or precentral cortex (P05), as well as in surrounding areas. The areas with higher SUVR in patient P05 were left hemisphere dominant, although such asymmetry was less apparent in patients P04, P06, or P07. None of the patients categorized as tau-negative by our visual read and ROI-based quantitative assessments by Z-score map (P08-P11) showed regions with significantly higher SUVR. In three Aβ-positive patients with bvFTD (P13-P15), significantly higher SUVR values were observed in widespread cortical regions to various degrees.

After applying a cluster-level threshold of $pFWE < 0.05$, several regions with higher SUVR remained significant in P02, P03, P05, P14, and P15, while no regions remained significant in P01, P04, P06, P07, and P13, presumably due to the small sample size of the control group.

2) Patients with other FTD phenotypes

In patients with other FTD phenotypes, one PPA patient (PPA1), three of four PNFA patients (PNFA1, PNFA2, PNFA4), and all CBS patients (CBS1-CBS3) showed regions with significantly higher SUVR values as compared to the controls, with both voxel-level height threshold of $p < 0.001$, uncorrected (> 20 voxels), and cluster-level threshold of $pFWE < 0.05$. Other patients (PPA2, PPA3, PNFA3, and PSP1) did not present cortical regions with significantly higher SUVR values with either of the statistical thresholds compared to the controls.

As shown in Supplementary Figure 2, patient PPA1 demonstrated significantly higher SUVR in relatively widespread regions in the inferior frontal and inferior and middle temporal cortices. In three of four PNFA patients (PNFA1, PNFA2, and PNFA4), significantly higher SUVR values were observed in the precentral cortex and/or parietal GM and WM regions. The regions with higher SUVR values in patient PNFA1 exhibited left-hemisphere dominance, while those in patient PNFA2 showed right-hemisphere dominance. On the other hand, no clear hemispheric dominance was found in patient PNFA4. Two Aβ-negative CBS patients (CBS1 and CBS2) demonstrated significantly higher SUVR values in the precentral cortex and surrounding WM regions, with left-hemisphere dominance. The Aβ-positive patient with CBS (CBS3) showed significantly higher SUVR values in widespread cortical regions.

3) Subgroups of 3RT-like and 4RT-like bvFTD patients as compared to controls

As shown in Supplementary Figure 3, 3RT-like patients with bvFTD (P01-P03) showed significantly higher SUVR in the bilateral orbital gyri and inferior and middle

frontal GM and nearby WM regions than the controls. In contrast, 4RT-like patients with bvFTD (P04-P07) showed significantly higher SUVR mainly in the bilateral superior parietal lobules, right postcentral and angular gyri, nearby WM regions, and left middle frontal cortex.

In the 3RT-like bvFTD cases, regions encompassing the right orbital gyrus remained significant after applying a cluster-level threshold of pFWE < 0.05. Meanwhile, no regions remained significant with pFWE < 0.05 in the 4RT-like bvFTD patients, presumably due to the focal distribution of tau lesions with little spatial overlap among the patients.

Supplementary Results 2

Associations between diagnostic confidence and tau topology subgroups in patients with bvFTD

Among the total 15 patients with bvFTD, 11, three, and one were classified as high, intermediate, and low diagnostic confidence, respectively.

The three patients with intermediate diagnostic confidence consisted of two Alzheimer's disease (AD)-like and one 4RT-like cases, while the one patient with low diagnostic confidence was classified as AD-like, according to our assessments of the ^{18}F-florzolotau accumulation pattern. The proportion of each putative pathological background by our ^{18}F-florzolotau assessments is shown in Supplementary Figure 4, for the total bvFTD sample and for the high diagnostic confidence of bvFTD, respectively.

As shown in Supplementary Figure 4, the most notable numerical difference between the two groups was the proportion of AD-like cases, which was 27% (four of 15 cases) in the total sample of bvFTD, and 9% (one of 11 cases) in the high confidence group.

Supplementary Figures

Supplementary Figure 1 Heatmaps of Z-scores for regional ^{18}F-florzolotau SUVRs in patients with non-behavioral-variant FTD phenotypes (n = 15)

Classification by visual read of tau PET images (see Figure 2) is indicated at the top. The left panel displays uncategorized Z-scores. Values exceeding the lower and upper limits are shown in blue and red, respectively. The right map presents the following Z-score ranges: light blue, < 1; orange, 1 – 2; red, > 2. CBS3 is Aβ-positive, and other patients are Aβ-negative.

Abbreviations: Aβ, amyloid-β; AD, Alzheimer's disease; CBS, corticobasal syndrome; GM, gray matter; PNFA, progressive non-fluent aphasia; PPA, primary progressive aphasia; PSP, progressive supranuclear palsy; SUVR, standardized uptake value ratio; WM, white matter; 3RT, three-repeat tau isoform; 4RT, four-repeat tau isoform

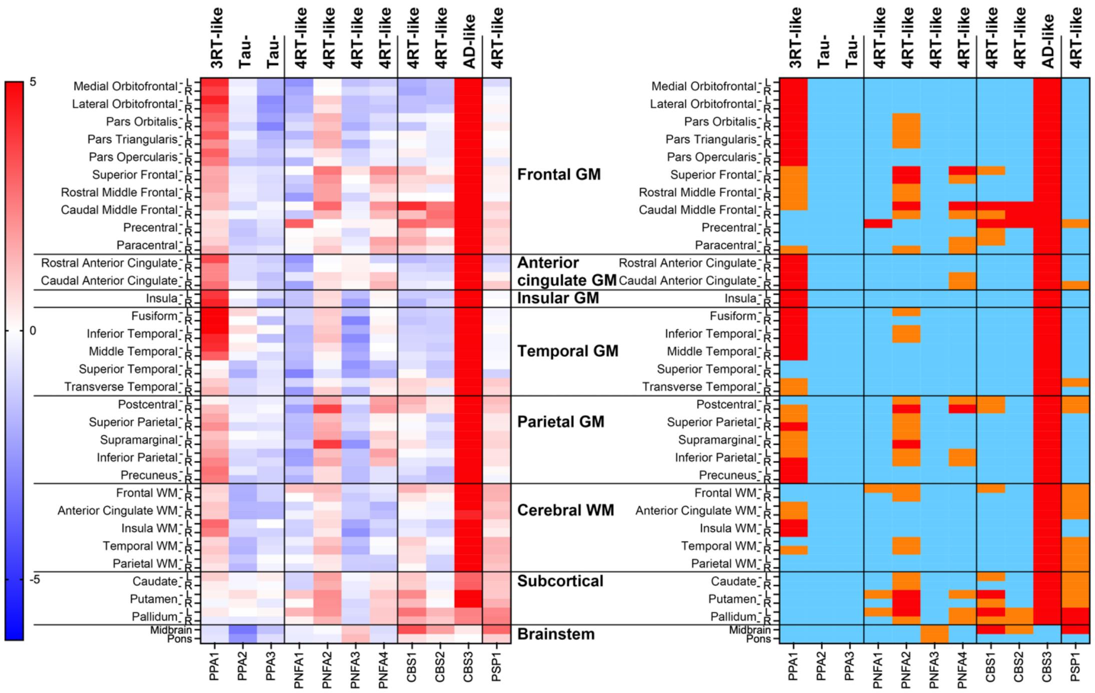

Supplementary Figure 2 Results of voxel-based analysis at an individual patient level showing regions with higher ^{18}F-florzolotau SUVR as compared to the controls

For illustrative purposes, results with the following two threshold conditions are displayed for each patient: (1) a voxel-level height threshold of $p < 0.001$, uncorrected, and extent of > 20 voxels (left); and (2) $p < 0.01$, uncorrected, (right). Results of patients are not displayed if there are no significant areas in the analysis with the first threshold condition. Analysis was restricted to gray and white matter of the frontal, temporal, and parietal cortices. Cases P01-P15, PPA1, PNFA1-PNFA4, and CBS1-CBS3 were patients with bvFTD, PPA, PNFA, and CBS, respectively. Patients P13-P15 and CBS3 were Aβ-positive.

Abbreviations: Aβ, amyloid-β; bvFTD, behavioral variant frontotemporal dementia; CBS, corticobasal syndrome; PNFA, progressive non-fluent aphasia; PPA, primary progressive aphasia; SUVR, standardized uptake value ratio

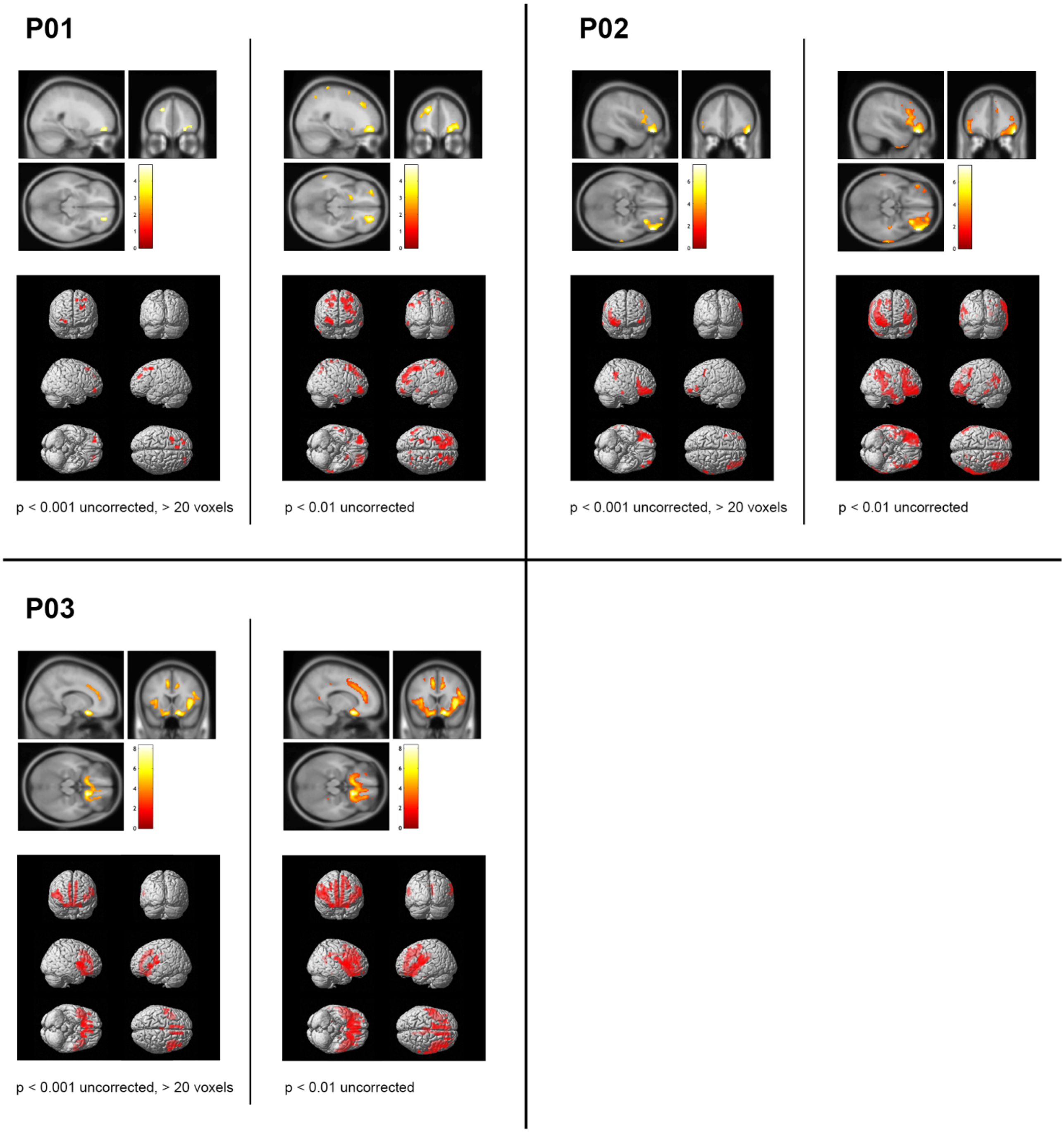
P01
p < 0.001 uncorrected, > 20 voxels
p < 0.01 uncorrected
P02
p < 0.001 uncorrected, > 20 voxels
p < 0.01 uncorrected
P03
p < 0.001 uncorrected, > 20 voxels
p < 0.01 uncorrected

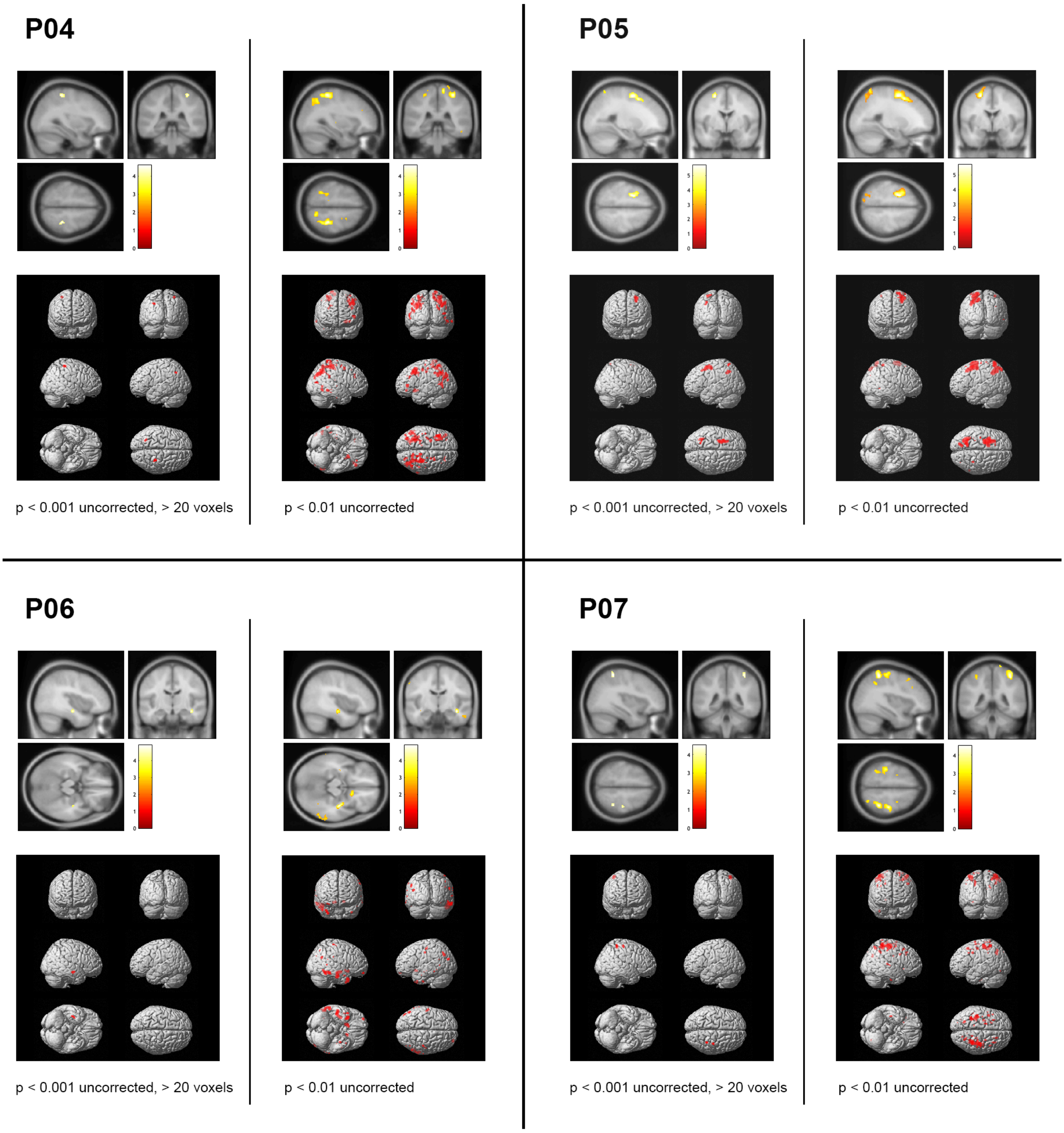
P04
p < 0.001 uncorrected, > 20 voxels
p < 0.01 uncorrected
P05
p < 0.001 uncorrected, > 20 voxels
p < 0.01 uncorrected
P06
p < 0.001 uncorrected, > 20 voxels
p < 0.01 uncorrected
P07
p < 0.001 uncorrected, > 20 voxels
p < 0.01 uncorrected

P13

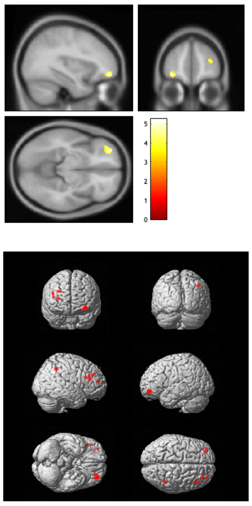

p < 0.001 uncorrected, > 20 voxels

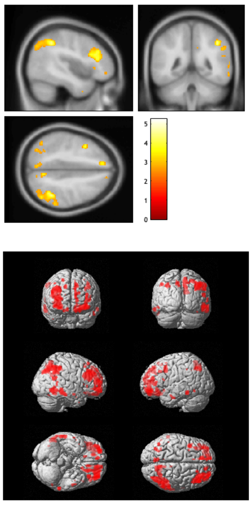

p < 0.01 uncorrected

P14

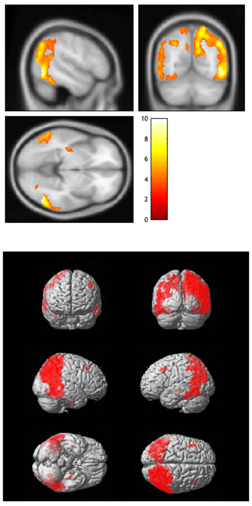

p < 0.001 uncorrected, > 20 voxels

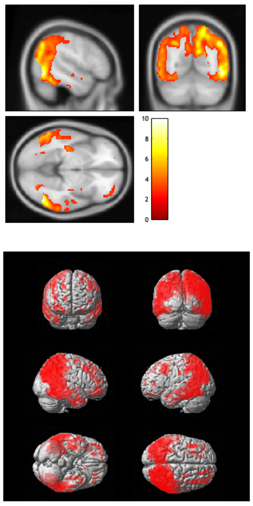

p < 0.01 uncorrected

P15

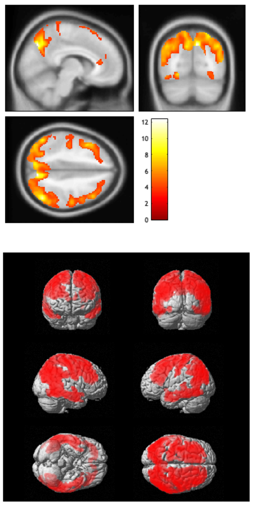

p < 0.001 uncorrected, > 20 voxels

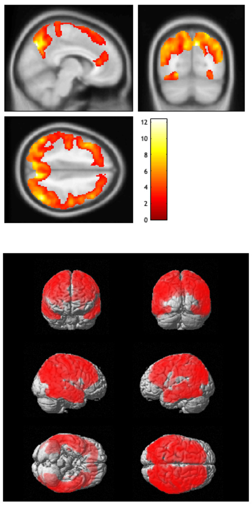

p < 0.01 uncorrected

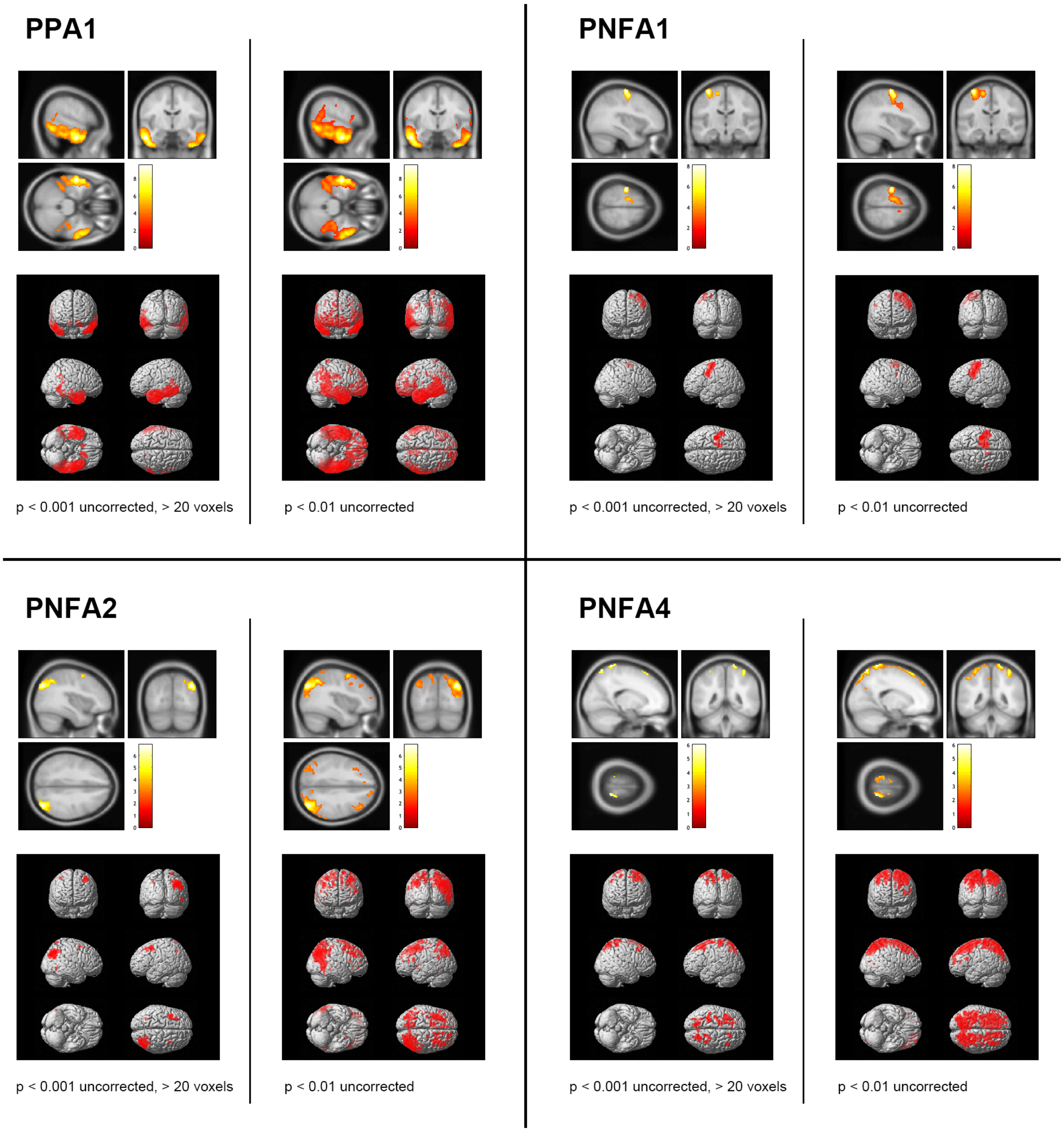
PPA1
p < 0.001 uncorrected, > 20 voxels
p < 0.01 uncorrected
PNFA1
p < 0.001 uncorrected, > 20 voxels
p < 0.01 uncorrected
PNFA2
p < 0.001 uncorrected, > 20 voxels
p < 0.01 uncorrected
PNFA4
p < 0.001 uncorrected, > 20 voxels
p < 0.01 uncorrected

CBS1

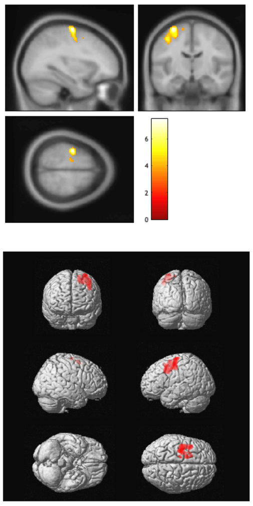

$p < 0.001$ uncorrected, > 20 voxels

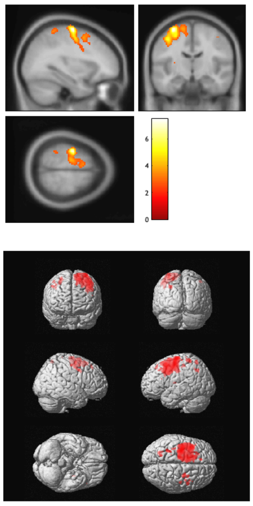

$p < 0.01$ uncorrected

CBS2

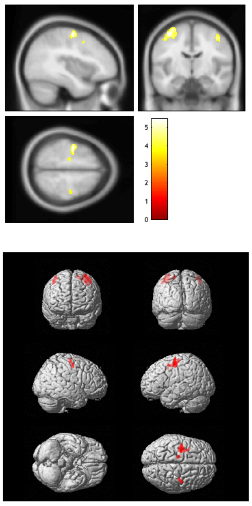

$p < 0.001$ uncorrected, > 20 voxels

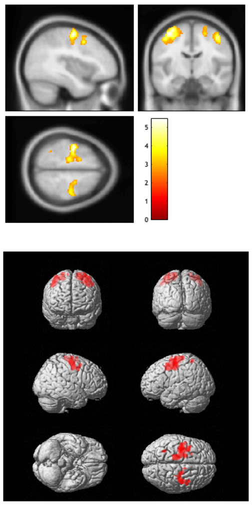

$p < 0.01$ uncorrected

CBS3

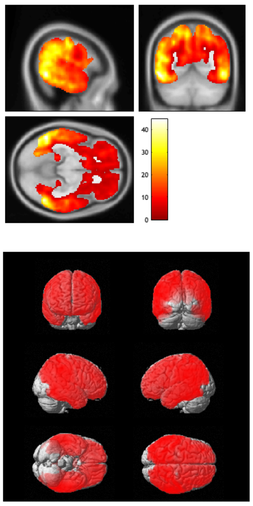

$p < 0.001$ uncorrected, > 20 voxels

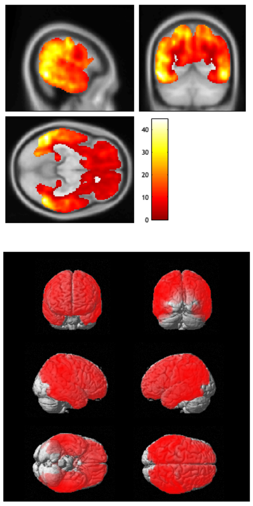

$p < 0.01$ uncorrected

Supplementary Figure 3 Voxel-wise group comparisons to identify regions with significantly higher SUVR in 3RT-like (P01-P03) and 4RT-like (P04-P07) bvFTD cases than 20 controls

For illustrative purposes, results with the following two threshold conditions are displayed: (1) a voxel-level height threshold of $p < 0.001$, uncorrected, and extent of > 20 voxels (left); and (2) $p = 0.01$, uncorrected, (right). The analysis was restricted to gray and white matter of the frontal, temporal, and parietal cortices.

Abbreviations: bvFTD, behavioral variant frontotemporal dementia; SUVR, standardized uptake value ratio; 3RT, three-repeat tau isoform; 4RT, four-repeat tau isoform

3RT-like bvFTD cases versus controls

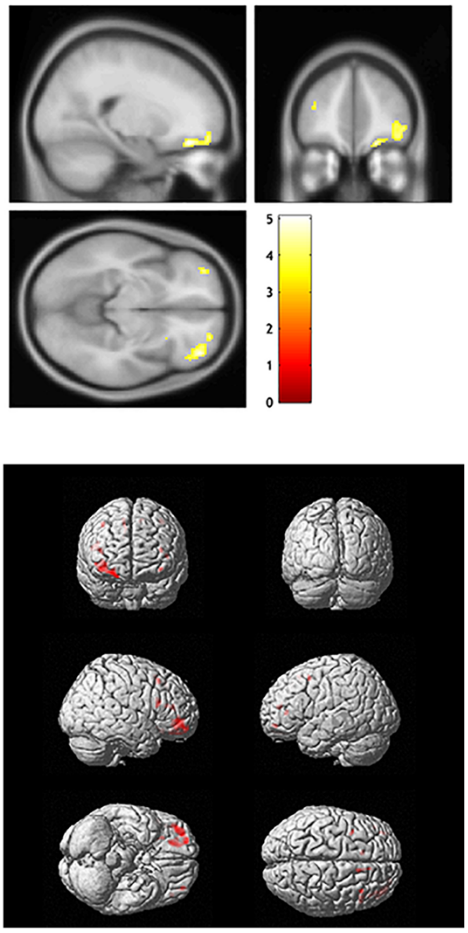

p < 0.001 uncorrected, > 20 voxels

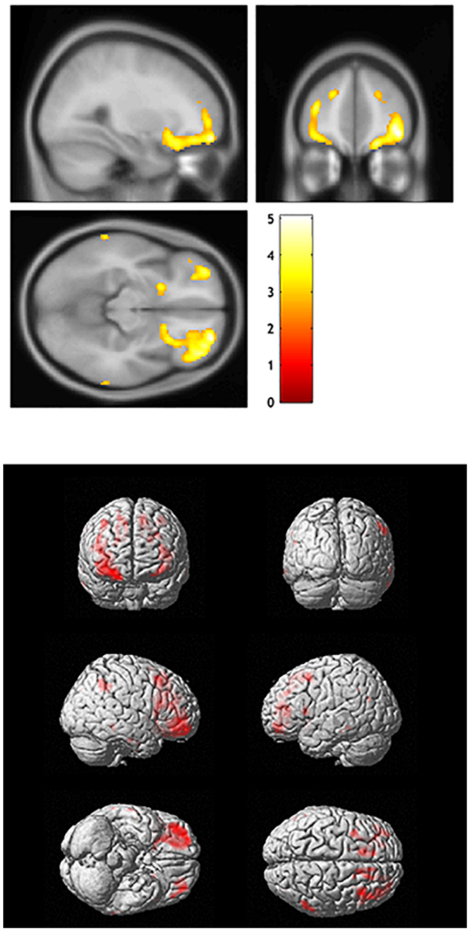

p < 0.01 uncorrected

4RT-like bvFTD cases versus controls

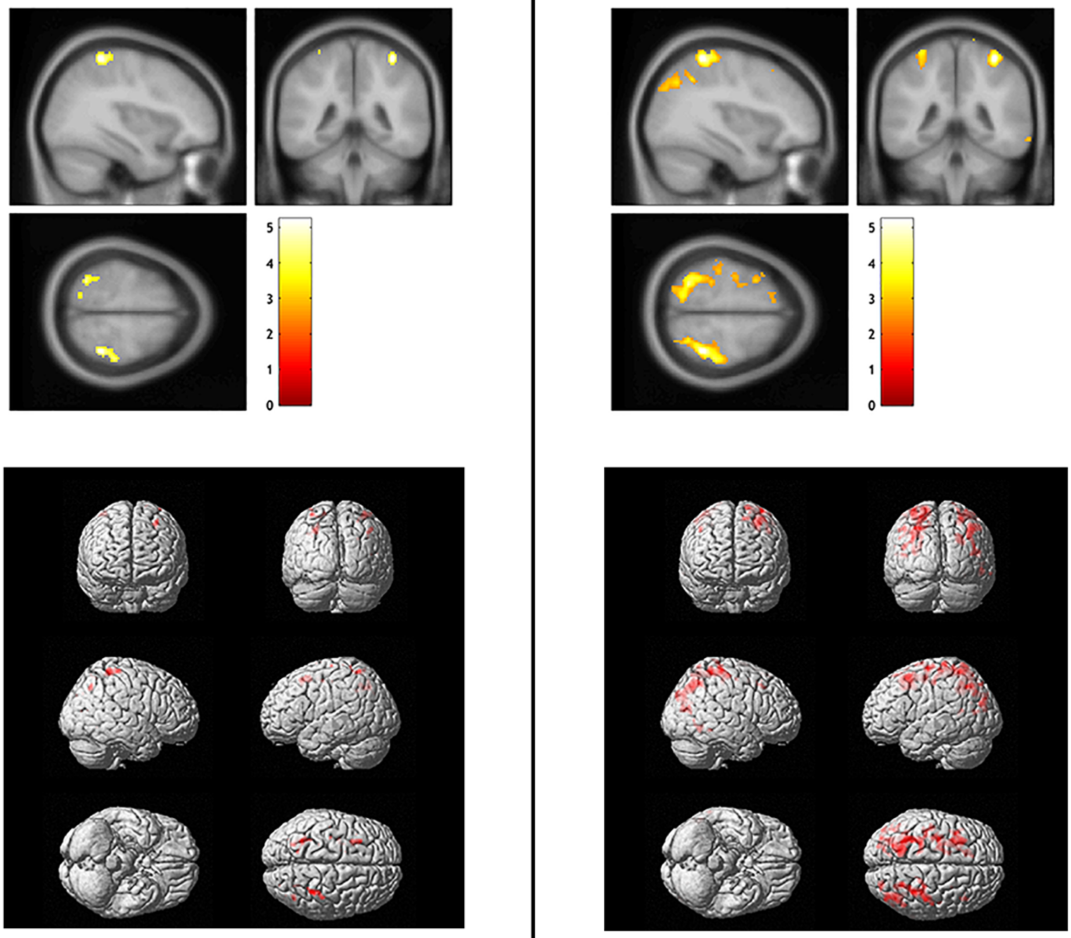

$p < 0.001$ uncorrected, > 20 voxels

$p < 0.01$ uncorrected

Supplementary Figure 4 Tau pathology classifications of bvFTD cases based on ^{18}F-florzolotau-PET findings for (A) all participants and for (B) those clinically diagnosed with high certainty

Abbreviations: AD, Alzheimer's disease; bvFTD, behavioral variant frontotemporal dementia; 3RT, three-repeat tau isoform; 4RT, four-repeat tau isoform

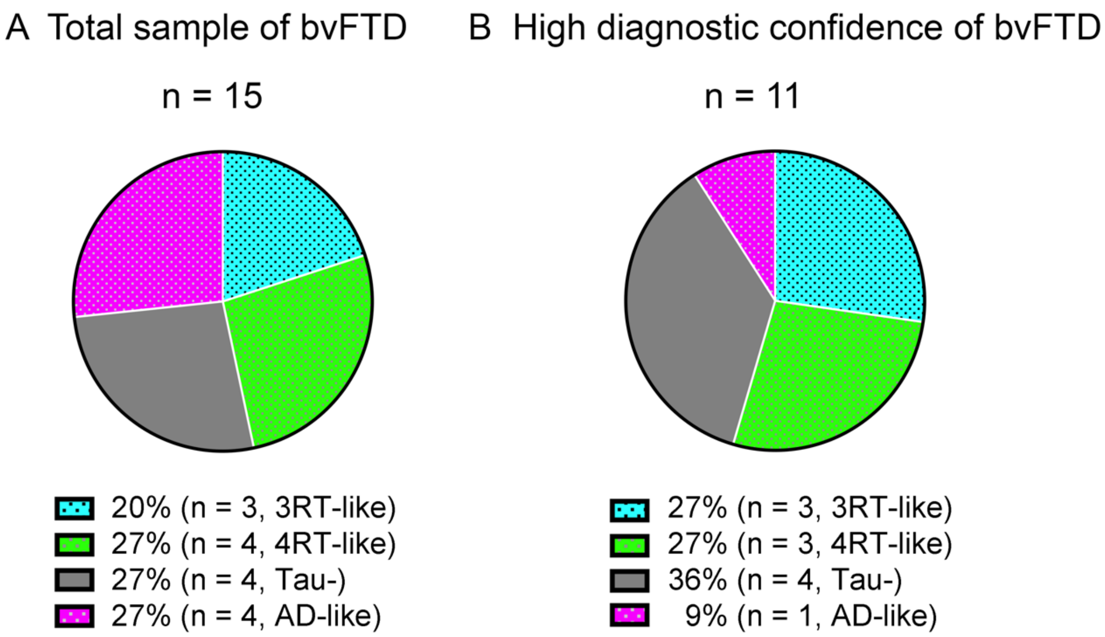

Supplementary Tables

Supplementary Table 1. Initial clinical symptoms and current neurological findings (presence or absence of parkinsonism) of patients in each bvFTD subgroup classified by our ^{18}F-florzolotau assessments

	Subgroup 1 (3RT-like)	Subgroup 2 (4RT-like)	Subgroup 3 (Tau-)	Subgroup 4 (AD-like)
**Number of patients**	3	4	4	4
**Initial symptoms**				
**Amnestic symptoms**	1	1	1	1
**Depressive symptoms**	0	1	3	1
**Manic and depressive symptoms**	0	0	0	1
**Hypochondriac symptoms**	0	1	0	0
**Persecutory delusion**	0	0	0	1
**Disinhibition**	2			
**Abnormal eating behavior**	0	1	0	0
**Current parkinsonism (Y/N)**	1/2	1/3	0/4	1/3

Abbreviations: AD, Alzheimer's disease; bvFTD, behavioral variant frontotemporal dementia; 3RT, three-repeat tau isoform; 4RT, four-repeat tau isoform

Supplementary References

1. Muller-Gartner HW, Links JM, Prince JL, et al. Measurement of radiotracer concentration in brain gray matter using positron emission tomography: MRI-based correction for partial volume effects. *J Cereb Blood Flow Metab*. Jul 1992;12(4):571-83. doi:10.1038/jcbfm.1992.81

2. Perry DC, Brown JA, Possin KL, et al. Clinicopathological correlations in behavioural variant frontotemporal dementia. *Brain*. Dec 1 2017;140(12):3329-3345. doi:10.1093/brain/awx254
